# *Mycobacterium tuberculosis* Immune Response in Patients With Immune-Mediated Inflammatory Disease

**DOI:** 10.3389/fimmu.2021.716857

**Published:** 2021-08-10

**Authors:** Elisa Petruccioli, Linda Petrone, Teresa Chiacchio, Chiara Farroni, Gilda Cuzzi, Assunta Navarra, Valentina Vanini, Umberto Massafra, Marianna Lo Pizzo, Giuliana Guggino, Nadia Caccamo, Fabrizio Cantini, Fabrizio Palmieri, Delia Goletti

**Affiliations:** ^1^Translational Research Unit, National Institute for Infectious Diseases Lazzaro Spallanzani-IRCCS, Rome, Italy; ^2^Clinical Epidemiology Unit, National Institute for Infectious Diseases Lazzaro Spallanzani-IRCCS, Rome, Italy; ^3^UOS Professioni Sanitarie Tecniche, National Institute for Infectious Diseases Lazzaro Spallanzani-IRCCS, Rome, Italy; ^4^Department of Internal Medicine, S. Pietro Fatebenefratelli Hospital, Rome, Italy; ^5^Department of Health Promotion, Mother and Child Care, Internal Medicine and Medical Specialties, Rheumatology Section-University of Palermo, Palermo, Italy; ^6^Central Laboratory of Advanced Diagnosis and Biomedical Research, University of Palermo, Palermo, Italy; ^7^Department of Biomedicine, Neurosciences and Advanced Diagnostic, University of Palermo, Palermo, Italy; ^8^Rheumatology Department, Hospital of Prato, Azienda USL Toscana Centro, Prato, Italy; ^9^Respiratory Infectious Diseases Unit, National Institute for Infectious Diseases Lazzaro Spallanzani-IRCCS, Rome, Italy

**Keywords:** tuberculosis, immune-mediated inflammatory disease, *M. tuberculosis*, IFN-γ, CD27

## Abstract

Subjects with immune-mediated inflammatory diseases (IMID), such as rheumatoid arthritis (RA), have an intrinsic higher probability to develop active-tuberculosis (TB) compared to the general population. The risk ranges from 2.0 to 8.9 in RA patients not receiving therapies. According to the WHO, the RA prevalence varies between 0.3% and 1% and is more common in women and in developed countries. Therefore, the identification and treatment of TB infection (TBI) in this fragile population is important to propose the TB preventive therapy. We aimed to study the M. tuberculosis (Mtb) specific T-cell response to find immune biomarkers of Mtb burden or Mtb clearance in patients with different TB status and different risk to develop active-TB disease. We enrolled TBI subjects as example of Mtb-containment, the active-TB as example of a replicating Mtb status, and the TBI-IMID as fragile population. To study the Mtb-specific response in a condition of possible Mtb sterilization, we longitudinally enrolled TBI subjects and active-TB patients before and after TB therapy. Peripheral blood mononuclear cells were stimulated overnight with Mtb peptides contained in TB1- and TB2-tubes of the Quantiferon-Plus kit. Then, we characterized by cytometry the Mtb-specific CD4 and CD8 T cells. In TBI-IMID, the TB therapy did not affect the ability of CD4 T cells to produce interferon-γ, tumor necrosis factor-α, and interleukin-2, their functional status, and their phenotype. The TB therapy determined a contraction of the triple functional CD4 T cells of the TBI subjects and active-TB patients. The CD45RA^-^ CD27^+^ T cells stood out as a main subset of the Mtb-specific response in all groups. Before the TB-preventive therapy, the TBI subjects had higher proportion of Mtb-specific CD45RA^-^CD27^+^CD4^+^ T cells and the active-TB subjects had higher proportion of Mtb-specific CD45RA^-^CD27^-^CD4^+^ T cells compared to other groups. The TBI-IMID patients showed a phenotype similar to TBI, suggesting that the type of IMID and the IMID therapy did not affect the activation status of Mtb-specific CD4 T cells. Future studies on a larger and better-stratified TBI-IMID population will help to understand the change of the Mtb-specific immune response over time and to identify possible immune biomarkers of Mtb-containment or active replication.

## Introduction

*M. tuberculosis* (Mtb) was estimated to be responsible for 10 million of new active tuberculosis (TB) disease cases and about 1.4 million TB deaths in 2019 (1). Almost a quarter of the world population is estimated to have TB infection (TBI) (2). However, only 5–10% of individuals with TBI will progress to active-TB disease during their lifetime (3). TB disease risk progression is higher within the first 2 years after Mtb exposure (3–6) and is strictly dependent on the efficiency of the immune system to control Mtb.

Indeed, among patients with the dysregulation of the immune system, subjects with immune-mediated inflammatory diseases (IMID), such as rheumatoid arthritis (RA), psoriatic arthritis (PsA), and ankylosing spondylitis (AS), have an intrinsic higher probability to develop active-TB compared to the general population (7–10). The risk ranges from 2.0 to 8.9 in RA patients not receiving therapies, and is lower in PsA and AS patients (7–11). The therapeutic strategy for IMID deeply changed thanks to the introduction of biologics drugs inhibiting specific pathway of the immune response. Briefly, we distinguish two main categories of biological drugs based on their action on tumor necrosis factor (TNF)-α: anti-TNF-α agents and non-anti-TNF-α agents. The anti-TNF-α agents, mainly but not exclusively monoclonal antibodies, include infliximab, adalimumab, golimumab, certolizumab pegol, and etanercept. The non-anti-TNF-α biologics include anti-Interleukin (IL)-1 anakinra, IL-6 inhibitor tocilizumab, anti-CD20 rituximab, anti-CD28 abatacept, anti-IL-12-23 Ustekinumab, anti-IL-17 secukinumab, and ixekizumab (10).

Recently, tofacitinib, baricitinib, upadacitinib, and filgotinib, a new class of synthetic drugs targeting the Janus kinases (JAKs) system, have been licensed for the treatment of rheumatoid arthritis (12). TB-reactivation risk associated with the JAK inhibitors seems negligible (13). However, since their recent marketing in Europe, these drugs were not investigated in the present study.

Among the different biologic agents, the anti-TNF-α increases the risk to develop TB disease up to 10 times (10). Indeed, TNF-α is fundamental for the Mtb-containment, inducing the construction and maintenance of the granuloma and stimulating the phagocytic ability of macrophages (10). Moreover, *in vitro* studies on Mtb-infected cells demonstrated that anti-TNF antibodies such as infliximab and adalimumab inhibit T-cell activation and IFN-γ production (14). Therefore, the risk related to Mtb-reactivation is associated with the direct effect of TNF-α blocking and to the indirect inhibition of other immune mediators. Differently, biologic drugs based on inhibition of IL-1, IL-6, IL-12-IL-23, IL-17, and CD28 lymphocytes act with less consequences on the granuloma integrity (11).

Therefore, the Mtb reactivation of IMID patients is due to both immune dysregulations related to the specific IMID and to the current therapeutic strategy.

Before starting the biologic therapy, based on these evidences, it is necessary to diagnose TBI among IMID patients and offer them the TB preventive therapy, preferably (11). Currently, the tuberculin skin test (TST) and the interferon-γ release assays (IGRAs), such as the QuantiFERON-TB Gold Plus (QFT-Plus) (Qiagen) and the T-Spot-TB (Oxford Immunotec), are the available commercial tests for detecting TBI (3, 15–18). Unfortunately, they have a poor predictive value for TB developing (3, 5, 15, 16, 18, 19). A recent meta-analysis reported that anti-TNF-α drugs significantly reduce the rate of positive score to IGRAs (20) and it has been demonstrated that IGRAs are falsely negative scored in RA patients with CD4 T-cell counts <650/μl and/or CD8 T-cell counts <400/μl (21). Moreover, we have recently demonstrated that that TBI-IMID had a lower IFN-γ response to QFT-Plus, with a higher proportion of results in the “uncertainty zone” (22) of QTF-plus assay compared to TBI individuals (23).

Several studies demonstrated that the TBI subjects with remote Mtb exposure, re-exposed to Mtb, had a lower probability to progress to active-TB disease compared to recently Mtb-infected subjects (4, 24), suggesting that the remote TBI achieved a sort of immune control of Mtb-infection. Probably in remote TBI individuals, Mtb remains in a low replication status continuously stimulating at low grade the immune system and favouring its containment. Indeed, several studies showed a decline of IFN-γ response levels during successful therapy, a condition of low or absent bacterial load (25–27). Due to the adult age of the manifestations of IMID, the majority of TBI-IMID subjects have a remote infection usually discovered during the TBI screening proposed before starting the biologics (28). Although a remote Mtb exposure, the TBI-IMID have an intrinsic higher risk to develop the active-TB disease (7, 10). In the last decade, several reports proposed that the differentiation status and functional ability of CD4 T cells depended on the degree and length of Mtb exposure (29, 30). Therefore, the different states of Mtb-infection could be identified by the differentiation status and functional ability of T cells (31, 32). Recently, it has been demonstrated that highly activated and moderately differentiated functional Mtb-specific T cells are potential immune biomarkers to discriminate recent and remote TBI individuals (29). Active-TB status has been associated to high level of cell-activation markers such as HLA-DR (33–37) or to the loss of CD27 (38–42).

In the last few years, several studies focused on the role of CD8 T cells in the Mtb-infection (32, 43–45). The CD8 T-cell response has been associated to Mtb load, showing that patients with active-TB and recent Mtb-infection have an increased Mtb-specific CD8 T-cell response (23, 25, 46–50). Moreover, decreased CD8 T-cell response during anti-TB treatment has been shown in longitudinal studies (25).

Currently, few reports are available on Mtb-specific immune response characterization in TBI-IMID. In this regard, Mtb-specific T cells producing IFN-γ, TNF-α, and IL-2 have been described in TBI-IMID patients under TNF-α antagonist therapy (51). Moreover, IFN-γ, IL-17, and IL-4 cytokines, which characterize three categories of differentiated CD4 T cells, may help distinguish the active-TB status from TBI-IMID with high specificity but low sensitivity (52).

Based on these findings, we aimed to study the Mtb-specific T-cell response to find immune biomarkers of Mtb burden or Mtb clearance in patients with different TB status and different risk to develop active-TB disease, such as the TBI-IMID individuals. We enrolled TBI subjects as a model for Mtb-containment, the active-TB as a model for replicating Mtb status, and the TBI-IMID as fragile population to contextualize it in the spectrum of TB. Moreover, to study the Mtb-specific response in a condition of possible Mtb sterilization, we longitudinally enrolled TBI subjects and active-TB patients before and after TB therapy.

## Material and Methods

### Population Characteristics

This study was approved by the Ethical Committee of “L. Spallanzani” National Institute of Infectious Diseases (INMI)-IRCCS, approval number 72/2015. Written informed consent was required to participate in the study conducted at INMI. We prospectively enrolled HIV-uninfected subjects with TBI with and without IMID and patients with pulmonary active-TB. Microbiologically diagnosed active-TB was defined based on the Mtb isolation from sputum culture. Active-TB patients were enrolled within 7 days of starting the specific TB treatment (T0) and at the end of therapy (T1).

In the absence of clinical, microbiological, and radiological signs of active-TB, TBI was defined based on a positive score to QFT-Plus (Qiagen, Hilden, Germany). The TBI cohort included subjects with a remote infection (contact with a smear-positive pulmonary TB patient at least 3 years before the enrolment) and subjects reporting a recent contact (within 3 months). TBI subjects reporting a time of exposure between 4 months and 3 years were not enrolled (38). TBI subjects and TBI-IMID patients were enrolled before starting the specific TB preventive therapy (T0) and at the end of treatment (T1). Demographic and epidemiological information were collected at enrolment and are reported in **Table 1**. The information relative to the type of IMID and IMID-therapy are reported in **Table 2**.

**Table 1:** Clinical characteristics of enrolled patients.

	TBI-IMID	TBI	Active-TB	TOTAL	p
**N**	14	12	13	39	
**Timing of TBI infection N (%):**					
** Recent**	2 (14)	8 (67)	NA	–	0.006*
** Remote**	12 (86)	4 (33)	NA	–	
**Age median (IQR)**	63 (54–75)	37 (22–53)	38 (26–42)	45 (29–56)	0.0002*
**Sex: Female N (%)**	6 (43)	9 (75)	6 (46)	21 (53)	0.2070**
**Origin N (%)**					
**West Europe**	10 (71.4)	6 (50)	6 (46)	22 (56)	
**East Europe**	2 (14.3)	5 (42)	5 (38)	12 (31)	
**Africa**	1 (7.1)	1 (8)	0 (0)	2 (5)	0.5295**
**Asia**	1 (7.1)	0 (0)	1 (8)	2 (5)	
**South America**	0 (0)	0 (0)	1 (8)	1 (3)	
**BCG vaccinated N (%)**	4 (28.5)	6 (50)	8 (61.5)	18 (46)	0.2175**
**Therapy N (%)**	14 (100) ^±^	12 (100)	13 (100) ^Š^	39 (100)	NA
**H**	11 (78.6)	8 (66.7)	–	18 (46.2)	
**H+R**	3 (21.4)	4 (33.3)	–	7 (17.9)	
**H+R+Z+E**	–	–	12 (92.3)	12 (30.7)	
**H+R+E+quinolone**	–	–	1 (7.7)	1 (2.6)	
**Therapy duration median (IQR)**	6 (4.6–6)	6 (3–6)	6 (6–9)	NA	NA
**QTF Plus N (%) at the time of enrolment** ^§^					0.4299**
**Positive**	12 (86)	11 (92)	12 (92)	34 (87)	
**Negative**	2 (14)	0 (8)	1 (8)	3 (77)	
**Number of lymphocytes x10^3^/μl at T0 median (IQR)** ^§§#^	1.9 (1.5–2.2)	2 (1.6–2.3)	1.7 (1–1.9)	1.8 (1.4–2.1)	0.13*
**Number of lymphocytes x10^3/^μl at T1 median (IQR)** ^§§§#^	1.6 (1.5–2.1)	1.9 (1.8–2.4)	2 (1.7–2.8)	1.9 (1.6–2.2)	0.051*
P values of the comparison of the number of lymphocytes x10^3^/μl at T1 *vs* the number of lymphocytes x10^3/^μl at T0 ^#^	0.75	0.73	0.002		

N, Number; TB, tuberculosis; TBI, TB infection; BCG, bacillus Calmette-Guérin; IQR, interquartile range; H, Isionazid; R, Rifampicin; Z, Pyrazinamide; E, Ethambutol; NA, not applicable *Kruskal- Wallis test; **Chi Square test; §All TBI and TBI-IMID patients have a QTF-Plus positive results in the past; ^±^One subject performed 2 weeks preventive therapy with H+R then continued with only H and therefore was included in the H-therapy group; ^Š^One patient discontinued and repeated the therapy.^§§^Data available at T0 (or within a month) in 14 TBI-IMID, 11 TBI, 13 Active-TB; ^§§§^Data available at T1 (or within a month) in 14 TBI-IMID, 9 TBI and 12 Active-TB; ^#^Wilcoxon matched-pairs signed rank test applied to compare T0 vs T1: TBI-IMID patients p = 0.75; TBI subjects p = 0.73; Active TB p = 0.002.

**Table 2:** Type of disease and type of therapy of TBI-IMID patients, enrolled before and after TB treatment.

Type of therapy	Psoriasis(2)		Psoriasic arthritis(2)		Rheumatoid arthritis(4)		Ankylosing spondylitis(6)		Total(14)	
	T0	T1	T0	T1	T0	T1	T0	T1	T0	T1
**Biologic, N**	0	0	0	0	0	1**	0	2^§,#^	0	3
**Biologic + CS, N**	0	0	0	0	0	1^+^	0	0	0	1
**Biologic +NSAID + ID, N**	0	0	0	1*	0	0	0	0	0	1
**Biologic + NSAID, N**	0	0	0	0	0	0	1^§^	0	1	0
**ID, N**	1	1	0	0	0	0	1	0	2	1
**NSAID, N**	0	0	1	1	1	1	1	1	3	3
**ID + CS + NSAID, N**	0	0	1	0	0	0	0	0	1	0
**ID + CS, N**	0	0	0	0	2	0	1	1	3	1
**ID + NSAID, N**	1	1	0	0	0	0	0	0	1	1
**No therapy, N**	0	0	0	0	1	1	2	2	3	3

T0, before TB therapy; T1, after TB therapy; N, Number; ID, Immunosuppressive Drug; CS, corticosteroids; NSAID, Nonsteroidal Anti-Inflammatory Drug; before *Ustekinumab (anti IL-12/IL-23),**Tocilizumab (anti-IL6); ^+^Abatacept (anti CTLA4); ^§^Golimumab (anti TNF-α), ^#^Etanercept (soluble receptor blocking the TNFα interaction with cell surface receptors).

### QFT-Plus Evaluation

QFT-Plus assay was performed for each patient at T0 and T1. Two TBI-IMID and one active-TB patients did not perform the T1 evaluation. QFT-Plus kits were used according to manufacturer’s instructions (53). The QFT-Plus Analysis Software (available from www.quantiFERON.com) was used to analyze raw data and to calculate the IFN-γ results in international units per milliliter (IU/ml). The software performs a quality control assessment of the assay, generates a standard curve, and provides a test result for each subject. Test results were interpreted according to manufacturer’s criteria (53). TB1 tube contained peptides designed to induce mainly a CD4 T-cell response, whereas TB2 tube contained peptides to induce both a CD4 and CD8 T-cell response (48, 49).

### Intracellular Staining Assay

Intracellular staining (ICS) was performed for each patient at T0 and T1. Peripheral blood mononuclear cells (PBMCs) were isolated using Ficoll density gradient centrifugation with the SepMate™ tubes (StemCell) and resuspended in complete RPMI-16-40 medium (Gibco, CA, USA) with 10% fetal bovine serum (PAA Laboratories GmbH, Pasching, Austria). To characterize by flow cytometry the Mtb-specific T-cell response, 1 × 10^6^ PBMC resuspended in 1 ml of medium were dispensed in TB1, TB2, Mitogen, and Nil tubes of the QFT-Plus kit. After a 1 h incubation, PBMCs were transferred in polystyrene round-bottom tubes, and 1 μl/ml of Golgi plug (BD Biosciences, San Josè, USA) was added to inhibit cytokine secretion. Anti-CD28 and anti-CD49d monoclonal antibodies (mAb) at 2 μg/ml each were added to co-stimulate cells.

Following an incubation of 16–24 h, the ICS was performed. As previously described (46), PBMCs were stained with anti-CD4 peridinin chlorophylprotein (PerCp)-Cy5.5 conjugate, anti-CD8 allophycocyanin (APC)-H7, anti-CD3 conjugate PE- cyanine 7 (Cy7), anti-IFN-γ Pacific Blue (PB) conjugate, anti-TNF-α fluorescein isothiocyanate (FITC), anti-IL-2 R-phycoerythrin (PE), anti-CD45RA APC, anti-CD27 Horizon V500 (all from BD Biosciences).

### Flow Cytometry Data Analysis

**Figure 1:**
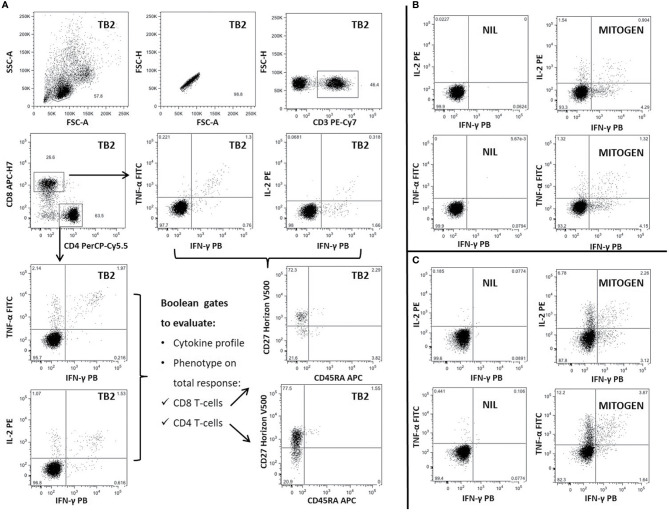
Gating strategy and representative panel of Mtb-specific CD4 and CD8 T-cell response in one patient with TBI-IMID. Briefly, lymphocytes were gated according to FSC and SSC parameters, doublets were excluded (FSC-A/FSC-H), and CD4 and CD8 T cells were gated inside the CD3-T cells subset. Frequency of IFN-γ, IL-2, and TNF-α producing T cells was evaluated inside CD4 and CD8 subsets. CD45RA and CD27 proportion on Mtb-specific T cells was evaluated inside the total cytokine response. The CD45RA and CD27 gating position was decided on bulk CD4 and CD8 T-cell subsets. **(A)** TB2-specific CD4 and CD8 T cells; **(B)** Cytokine panels of CD8 T cells stimulated with NIL and MITOGEN; **(C)** Cytokine panels of CD4 T cells stimulated with NIL and MITOGEN. FSC, forward scatter; SSC, side scatter; TB2, peptides of TB2 tube of QFT-plus kit; MITOGEN, positive control stimulation of QFT-plus kit; NIL, unstimulated tube of QFT-plus kit.

The Mtb-specific T-cell response was characterized evaluating the frequencies of CD4 and CD8 T cells producing IFN-γ, TNF-α, and IL-2 (**Figure 1**). At least 100,000 lymphocytes were acquired with a FACS CANTO II (BD Biosciences). Cytometry data were analyzed using FlowJo software (Version 9.3). Background cytokine production in the Nil tube was subtracted from each stimulated condition. If the background was higher than half of the antigen-specific response, the results were scored as negative. A frequency of IFN-γ-producing T cells of at least 0.03% was considered as positive response. The cytokine profile has been evaluated only in patients with a positive total response to the Mtb stimulation using the Boolean gate function of FlowJo software. For responders, we also calculated the functional differentiation score (FDS) as previously described (29, 30) by applying the following equation:

$$\text{FDS}=\frac{\text{IFN}-\gamma^{+}\;\text{IL}-2^{+/-}\;\text{TNF}-{\text{α}}^{+/-}}{\text{IFN}-\gamma^{-}\;\text{IL}-2^{+/-}\;\text{TFN}-{\text{α}}^{+/-}}$$

The phenotype as well has been evaluated only in responders, assessing the proportion of CD45RA and CD27 on the gate of CD4 T cells able to produce IFN-γ, IL-2, or TNF-α (total CD4 T-cell response).

The number of CD4 and CD8 responders to TB1 and TB2 stimulation is reported in detail in **Table 3**.

**Table 3:** Number of CD4 and CD8 T-cell responders among IMID-TBI, TBI subjects, and active-TB patients, enrolled before and after TB treatment.

Stimulation and type of T-cell response	Type of cytokines produced	TBI-IMID(14)		TBI(12)		Active-TB(13)		p*	
		T0 N	T1 N	T0 N	T1 N	T0 N	T1 N	T0	T1
**TB1 specific CD4 T cells**	**Any cytokines**	13 (93)	12 (86)	11 (92)	10 (83)	10 (77)	10 (77)	0.493	0.878
	**IFN-γ**	12 (86)	10 (71)	9 (75)	8 (67)	9 (69)	8 (61)	0.641	0.913
	**TNF-α**	11 (78)	11 (78)	11 (92)	8 (67)	10 (77)	9 (69)	0.665	0.822
	**IL-2**	10 (71)	11 (78)	10 (83)	8 (67)	7 (54)	7 (54)	0.290	0.436
**TB2 specific CD4 T cells**	**Any cytokines**	11 (78)	12 (86)	12 (100)	11 (92)	11 (85)	11 (85)	0.340	1.000
	**CD4 IFN-γ**	11 (78)	12 (86)	10 (83)	10 (83)	10 (77)	9 (69)	1.000	0.610
	**TNF-α**	10 (71)	12 (86)	12 (100)	9 (75)	9 (69)	9 (69)	0.094	0.641
	**IL-2**	9 (64)	9 (64)	10 (83)	7 (58)	6 (46)	8 (61)	0.198	1.000
**TB1 specific CD8 T cells**	**Any cytokines**	3 (21)	4 (28)	4 (33)	6 (50)	3 (23)	5 (38)	0.811	0.552
	**IFN-γ**	3 (21)	4 (28)	4 (33)	6 (50)	3 (23)	5 (38)	0.811	0.552
	**TNF-α**	0 (0)	0 (0)	1 (8)	0 (0)	0 (0)	2 (15)	0.308	0.194
	**IL-2**	0 (0)	1 (7)	1 (8)	2 (17)	1 (8)	2 (15)	0.528	0.716
**TB2 specific CD8 T cells**	**Any cytokines**	2 (14)	4 (28)	4 (33)	8 (67)	4 (30)	6 (46)	0.481	0.160
	**CD4 IFN-γ**	2 (14)	4 (28)	4 (33)	8 (67)	4 (31)	5 (38)	0.481	0.145
	**TNF-α**	0 (0)	1 (7)	1 (8)	2 (17)	2 (15)	4 (31)	0.393	0.291
	**IL-2**	0 (0)	1 (7)	0 (0)	2 (17)	1 (8)	1 (8)	0.641	0.668

**T0**, before TB therapy; T1, after TB therapy; N, Number; *Fisher Test.

### Statistical Analysis

Data were analyzed using Graph Pad Prism (Version 8.2.1 for Windows) and Stata (Stata 15, StataCorp. 2017. Stata Statistical Software: Release 15. College Station, TX: StataCorp LLC). The median and interquartile ranges (IQRs) were calculated for continuous measures. Kruskal–Wallis test was used for comparison among several groups. The Chi-Square and Fisher tests were used for proportions. The Wilcoxon matched-pairs signed rank test was used to compare the different time points. Bonferroni correction was applied when appropriate.

## Results

### Characteristics of the Population

Thirty-nine subjects at different TB stages were enrolled. The TB-IMID patients had the higher age compared to the other groups (p = 0.0002), they included a higher proportion of remote Mtb exposed subjects compared to TBI (p=0.006), and they have a similar number of lymphocytes before and after TB therapy completion (p = 0.75, **Table 1**). One TBI-IMID patient was taking biologic drugs at T0 and at T1, and four TBI-IMID patients were taking biologics at T1 (**Tables 2**, **4**). Regarding the TB therapy, the majority of TBI-IMID patients received isionazid, whereas only three received isoniazid and rifampicin for 3 months; one subject received for 2 weeks isionazid and rifampicine then continued with isoniazid only. The duration of the preventive therapy regimen had a median of 6 months (**Table 1**). The TBI subjects showed similar lymphocyte counts before and after therapy (p = 0.73, **Table 1**) and received mainly isoniazid. Finally, as expected, patients with active-TB showed a significant increase of the lymphocyte counts after TB therapy completion (p = 0.002, **Table 1**) and received the standard TB regimen.

**Table 4:** Comparison of CD4 T-cells cytokine production according to the use of biologic drugs at T0 and T1.

Biologic agents	N	Any cytokine in response to TB1			Any cytokine in response to TB2		
		T0 mean (IQR)	T1 mean (IQR)	P*	T0 mean (IQR)	T1 mean (IQR)	P*
Never	9	0.31 (0.10–0.74)	0.41 (0.14–0.50)	0.722	0.17 (0–0.38)	0.43 (0.21–0.68)	0.094
Only T0	0	–	–	–	–	–	–
Only T1	4	0.35 (0.24–0.50)	0.26 (0.19–0.74)	0.715	0.39 (0.18–0.55)	0.50 (0.18–0.82)	0.273
T0 and T1	1	0.17 (na)	0.30 (na)	(na)	0.12 (na)	0.14 (na)	(na)
Overall	14	0.29 (0.17–0.54)	0.31 (0.18–0.50)	0.850	0.20 (0.9–0.55)	0.43 (0.15–0.80)	0.032

T0, before TB therapy; T1, after TB therapy; N, Number; *Wilcoxon matched-pairs signed rank test; na, not available.

### QFT-Plus Trend Before and After TB Treatment

In this study, the diagnosis of TBI is based on a positive IGRA and clinical and radiological parameters; therefore, the TBI-IMID and the TBI subjects have for definition a positive IGRA result. However, among the TBI-IMID patients, two patients had a negative QFT-Plus at T0 and a previously positive IGRA: one patient had a remote Mtb-infection, whereas the other one had a recent Mtb-infection and was taking an immunosuppressive drug. Among the subjects with TBI, one patient with a remote Mtb-infection had a negative QFT-Plus at T0 and a previously positive IGRA.

Differently, the assumption of a positive IGRA is not a criterion for the diagnosis of active-TB disease. Evaluating the QFT-Plus response at T0 and T1, we did not observe any significant differences in any groups (**Supplementary Figure 1**), and remarkably, all patients responded to Mitogen stimulation (data not shown).

### Frequency of Mtb-Specific T Cells Is Similar in Patients Enrolled Before and After TB Treatment

**Figure 2:**
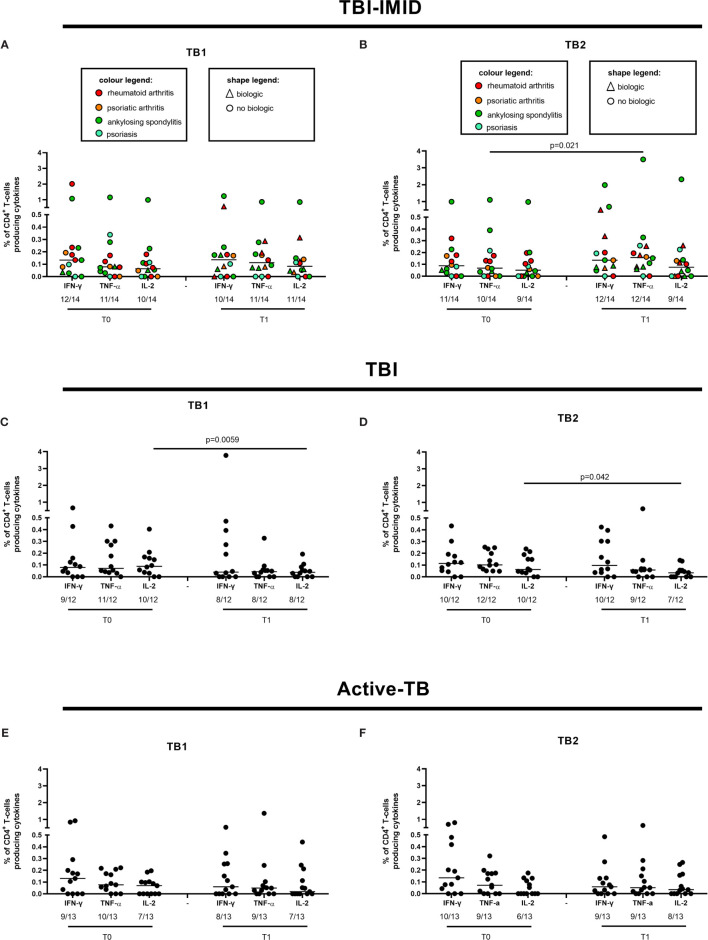
CD4 T cells producing IFN-γ, IL-2, and TNF-α in response to Mtb antigen stimulation before and after TB therapy completion. PBMC of patients enrolled before (T0) and after TB therapy (T1) were stimulated overnight with TB1 and TB2 peptides. **(A, B)** TBI-IMID; **(C, D)** TBI subjects; **(E, F)** Active-TB patients. Number of responders over total enrolled patients is reported below each panel. Horizontal lines indicate the median. Statistical analysis was performed using the Wilcoxon matched-pairs signed rank test and the p value was considered significant if ≤0.05. TB, tuberculosis; TBI, tuberculosis infection; IMID, immune mediated inflammatory disease; TB1, peptides of TB1 tube of QFT-plus kit; TB2, peptides of TB2 tube of QFT-plus kit.

**Figure 3:**
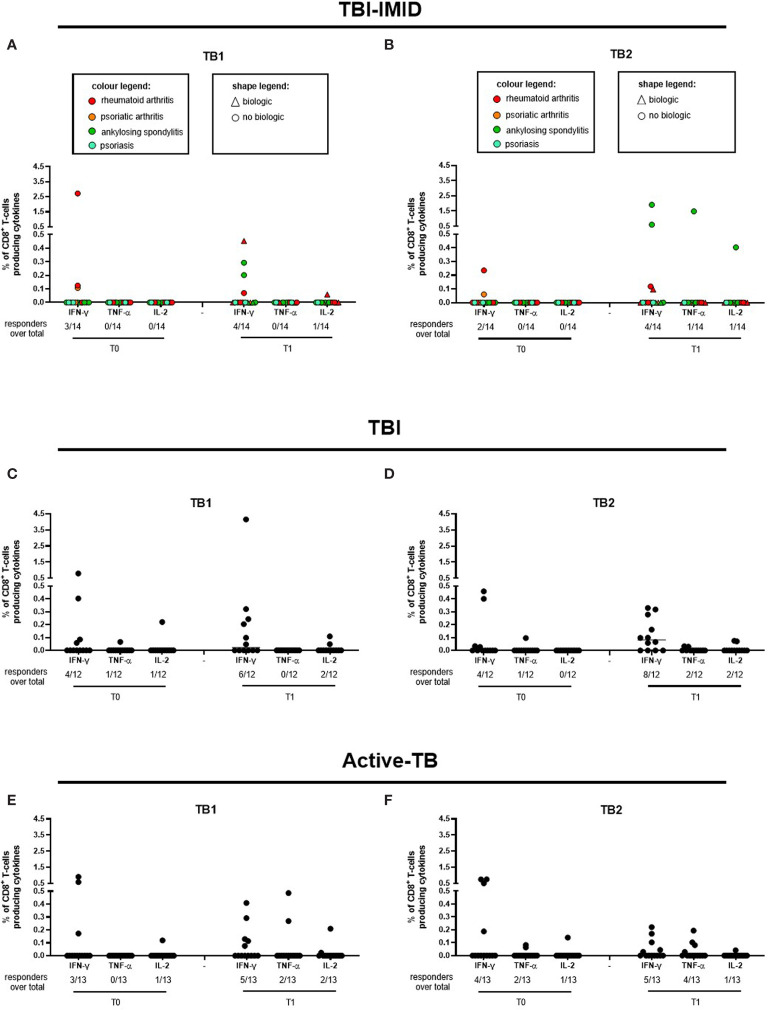
CD8 T cells producing IFN-γ, IL-2, and TNF-α in response to Mtb antigen stimulation. PBMC of patients enrolled before (T0) and after TB therapy (T1) were stimulated overnight with TB1 and TB2 peptides. **(A, B)** TBI-IMID; **(C, D)** TBI patients; **(E, F)** Active-TB patients. Number of responders over total enrolled patients is reported below each panel. Horizontal lines indicate the median. Statistical analysis was performed using the Wilcoxon matched-pairs signed rank test and the p value was considered significant if ≤0.05. TB, tuberculosis; TBI, tuberculosis infection; IMID, immune mediated inflammatory disease; TB1, peptides of TB1 tube of QFT-plus kit; TB2, peptides of TB2 tube of QFT-plus kit.

We evaluated the ability of CD4 and CD8 T cells to produce IFN-γ, IL-2, and TNF-α in response to TB1 or TB2 stimulation (**Figures 2**, **3** and **Table 3**). In **Figure 1**, we showed a representative flow-cytometry analysis of Mtb-specific T cells for each studied group. Regarding the Mtb-specific CD4 T-cell response, we did not observe significant differences in terms of number of responders before and after TB treatment in any groups (**Table 3**). The frequency of Mtb-specific CD4 T cells, producing IFN-γ, IL-2, and TNF-α, was similar among TBI-IMID (**Figures 2A, B**), TBI subjects (**Figures 2C, D**), and active-TB patients (**Figures 2E, F**) at T0 and T1 with the exception of a lower frequency of TB2-specific TNF-α^+^ CD4 T cells at T0 compared to T1 in TBI-IMID group (**Figure 2B**) and a higher frequency of TB1- and TB2-specific IL-2^+^ CD4 T cells at T0 compared to T1 in TBI group (**Figures 2C, D**). Moreover, all groups of patients produced IFN-γ, IL-2, and TNF-α in response to TB1 or TB2 stimulations (**Figure 2** and **Table 3**). Considering the different IMID therapy of TBI-IMID (**Table 2**), we indicated in the graph the presence or not of an undergoing therapy with biologic drugs at each time point (**Figures 2A, B**). Stratifying for the presence of the undergoing biologic therapy (**Table 4**), we found that four patients were taking biologics only at T1, one patient at both T0 and T1, and nine patients neither at T0 nor at T1. We did not find any significant differences among TBI-IMID comparing T0 and T1 in terms of total response (production of any cytokines by CD4 T cells) (**Table 4**). Due to sample size, we could not stratify for type of biologic agents used and for the others IMID therapies.

Regarding the Mtb-specific CD8 T-cell response, we observed a not significant higher number of responders at T1 compared to T0 in response to TB2 stimulation (**Table 3**). Moreover, the number of CD8 T-cell responders was lower than the number of CD4 T-cell responders (**Table 3**). The frequency of Mtb-specific CD8 T cells was similar among TBI-IMID (**Figures 3A, B**), TBI subjects (**Figures 3C, D**), and active-TB (**Figures 3E, F**) patients at T0 and T1 and the responders produced mainly IFN-γ in response to TB1 or TB2 stimulations. Due to the low number of CD8 T-cell responders, we did not analyze the data according to the presence or not of biologic drugs.

Patients of all studied groups responded to the *in vitro* mitogen stimulation (**Supplementary Figure 2**). Regarding the CD4 T-cell response, the TBI-IMID patients showed a higher frequency of T cells producing IFN-γ or TNF-α or IL-2 at T1 compared to T0, whereas no significant differences were observed in TBI subjects and active-TB patients (**Supplementary Figure 2A–E**). Regarding the CD8 T-cell response, the TBI subjects showed a higher frequency of T cells producing IL-2 at T0 compared to T1, whereas no significant differences were observed in TBI-IMID and active-TB patients (**Supplementary Figure 2B–F**).

**Figure 4:**
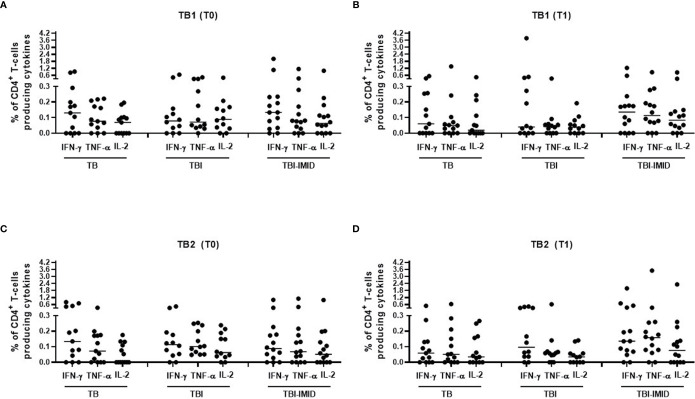
Comparison of CD4 T cells producing IFN-γ, IL-2, and TNF-α in response to Mtb antigen stimulation at each time point. PBMC of patients enrolled before (T0) and after TB therapy (T1) were stimulated overnight with TB1 and TB2 peptides. **(A, B)** TB1 response at T0 and T1; **(C, D)** TB2 response at T0 and T1. Horizontal lines indicate the median. Statistical analysis was performed using the Mann–Whitney unmatched test, Bonferroni correction was applied, and the p value was considered significant if ≤0.017. TB, tuberculosis; TBI, tuberculosis infection; IMID, immune mediated inflammatory disease; TB1, peptides of TB1 tube of QFT-plus kit; TB2, peptides of TB2 tube of QFT-plus kit.

We next compared the cytokine production of Mtb-specific CD4 T cells among the different groups at each time point (**Figure 4**); we focused on CD4 T cells since the number of CD8 T cells responders was too low to allow a robust statistical analysis (**Table 3**). The frequency of Mtb-specific CD4 T cells producing IFN-γ, IL-2, or TNF-α was similar in TB, TBI subjects, and TBI-IMID patients enrolled at T0 (**Figures 4A, C**) and at T1 (**Figures 4B, D**). Differently, in response to mitogen stimulation, we reported a higher level of TNF-α (p = 0.0013) and IL-2 in TBI-IMID compared to TBI (**Supplementary Figure 3**).

### TB-IMID Patients Had a Similar Cytokine Profile Before and After TB Preventive Therapy

**Figure 5:**
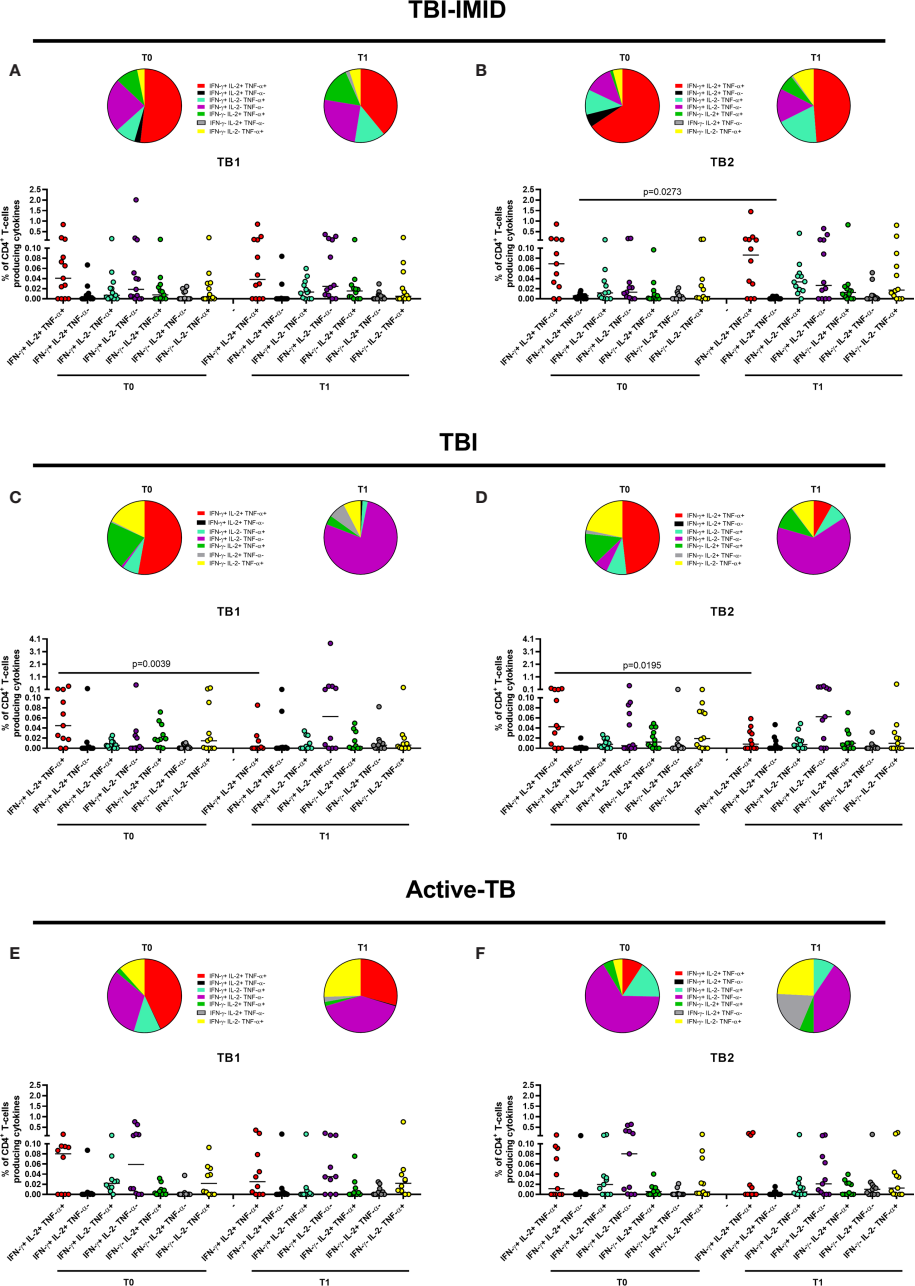
Functional profile of Mtb-specific CD4 T cells before and after TB therapy completion. PBMC of patients enrolled before (T0) and after TB therapy (T1) were stimulated overnight with TB1 and TB2 peptides. **(A, B)** TBI-IMID patients; **(C, D)** TBI subjects; **(E, F)** Active-TB patients. Cytokine profile was evaluated only on responders using Boolean gate combination. The number of CD4 and CD8 responders to TB1 and TB2 stimulation is reported in detail in **Table 3**. Horizontal lines indicate the median. Statistical analysis was performed using the Wilcoxon matched-pairs signed rank test, and the p value was considered significant if ≤0.05. TB, tuberculosis; TBI, tuberculosis infection; IMID, immune mediated inflammatory disease; TB1, peptides of TB1 tube of QFT-plus kit; TB2, peptides of TB2 tube of QFT-plus kit.

We further investigated the functional cytokine profile of Mtb-specific T cells (**Figure 5**). We focused on CD4 T cells since the number of CD8 T cells responders was too low to allow a robust statistical analysis (**Table 3**).

In TBI-IMID group, we did not observe any significant changes comparing CD4 cytokine profile at T0 and T1 (**Figures 5A, B**) with the exception of a decrease of the IFN-γ^+^ IL-2^+^ CD4 T-cell subset at T1 compared to T0 in response to TB2 stimulation (**Figure 5B**). The CD4 T-cell response to TB1 and TB2 was mainly characterized by polyfunctional IFN-γ^+^ IL-2^+^ TNF-α^+^ CD4 T cells; among the monofunctional T cells, the IFN-γ^+^ IL-2^-^ TNF-α^-^ CD4 T cells were the most representative subset (**Figures 5A, B**).

In TBI group, we observed significant changes comparing CD4 T-cell cytokine profile at T0 and T1 (**Figures 5C, D**): the proportion of polyfunctional IFN-γ^+^ IL-2^+^ TNF-α^+^ CD4 T cells was significantly higher at T0 than at T1 in response to TB1 (p = 0.0039) and to TB2 (p = 0.0195). Differently, we reported a higher but not significant proportion of monofunctional IFN-γ^+^ IL-2^-^ TNF-α^-^ CD4 T cells at T1 compared to T0 in response to both stimuli. The proportion of the monofunctional IFN-γ^+^ IL-2^-^ TNF-α^-^ T cells in TBI subjects after TB treatment was quite higher compared to TBI-IMID at the same time point (see below for the details).

In the active-TB group, we did not observe any significant change comparing CD4 cytokine profile at T0 and T1 (**Figures 5E, F**). In this case, the CD4 response to TB1 and TB2 was not similar. The TB1 response was mainly characterized by polyfunctional IFN-γ^+^ IL-2^+^ TNF-α^+^ CD4 T cells; among the monofunctional T cells, the IFN-γ^-^ IL-2^-^ TNF-α^+^ CD4 T cells and the IFN-γ^+^ IL-2^-^ TNF-α^-^ CD4 T cells were the most representative subsets (**Figure 5E**). In contrast, the TB2 response showed a low proportion of polyfunctional IFN-γ^+^ IL-2^+^ TNF-α^+^ CD4 T cells and an increased proportion of monofunctional IFN-γ^+^ IL-2^-^ TNF-α^-^ CD4 T cells at both time points (**Figure 5F**).

It is known that Mtb-specific T cells change their functional capacity depending on antigen and bacterial load (25, 30, 31, 33). Based on these evidences, it has been proposed a single measurement of functional differentiation, FDS, of responders that describes the different type of Mtb-infection and Mtb burden (30). This analysis led us to synthetize the functional changes of T cells before and after therapy reported in **Figure 5**. The FDS of Mtb-specific CD4 T cells before and after therapy was not significantly different between T0 and T1 in any groups of patients (**Supplementary Figure 4**). The FDS comparison at the baseline did not show any significant differences among groups (data not shown). To note that, we could not include in the FDS calculation the patients without the IFN-γ^-^ IL-2^+/-^ TNF-α^+/-^ CD4^+^ T-cell subset (TBI-IMID T0 in response to TB1: two patients, TBI-IMID T1 in response to TB1: one patient; TBI-IMID T0 in response to TB2: one patient; TBI T1 in response to TB2: one patient; active-TB T0 in response to TB2: two patients).

Since the TBI-IMID and the TBI included both recent and remote Mtb-infection, we choose to analyze again the FDS including the most represented category: remote TBI-IMID (12 patients over 14 enrolled) and recent TBI (8 patients over 12 enrolled) (**Supplementary Figure 5**). Even with this stratification, we did no observe significant differences before and after TB therapy in any studied groups.

Collectively, these data suggested that the TB treatment did not deeply affect the CD4 cytokine profile of the TBI-IMID patients. Differently, the TBI subjects showed a significant decrease of triple functional T cells after treatment and the active-TB a not significant but evident increase of the proportion of monofunctional IFN-γ^+^ IL-2^-^ TNF-α^-^ CD4^+^ T cells (**Figure 5**).

**Figure 6:**
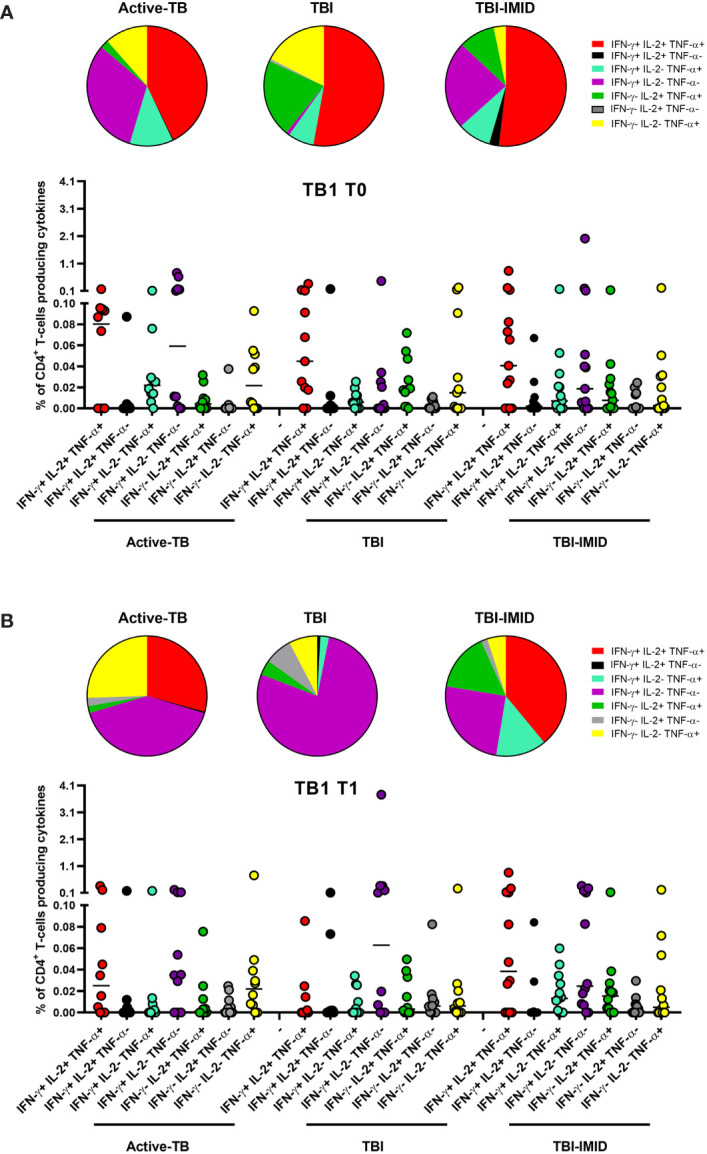
Comparison of functional profile of TB1 specific CD4 T cells at each time point. PBMC of patients enrolled before (T0) and after TB therapy (T1) were stimulated overnight with TB1 peptides. **(A)** T0; **(B)** T1. Cytokine profile was evaluated only on responders using Boolean gate combination. The number of CD4 and CD8 responders to TB1 and TB2 stimulation is reported in detail in **Table 3**. Horizontal lines indicate the median. Statistical analysis was performed using the Mann–Whitney unmatched test, Bonferroni correction was applied, and the p value was considered significant if ≤0.017. TB, tuberculosis; TBI, tuberculosis infection; IMID, immune mediated inflammatory disease; TB1, peptides of TB1 tube of QFT-plus kit; TB2, peptides of TB2 tube of QFT-plus kit.

**Figure 7:**
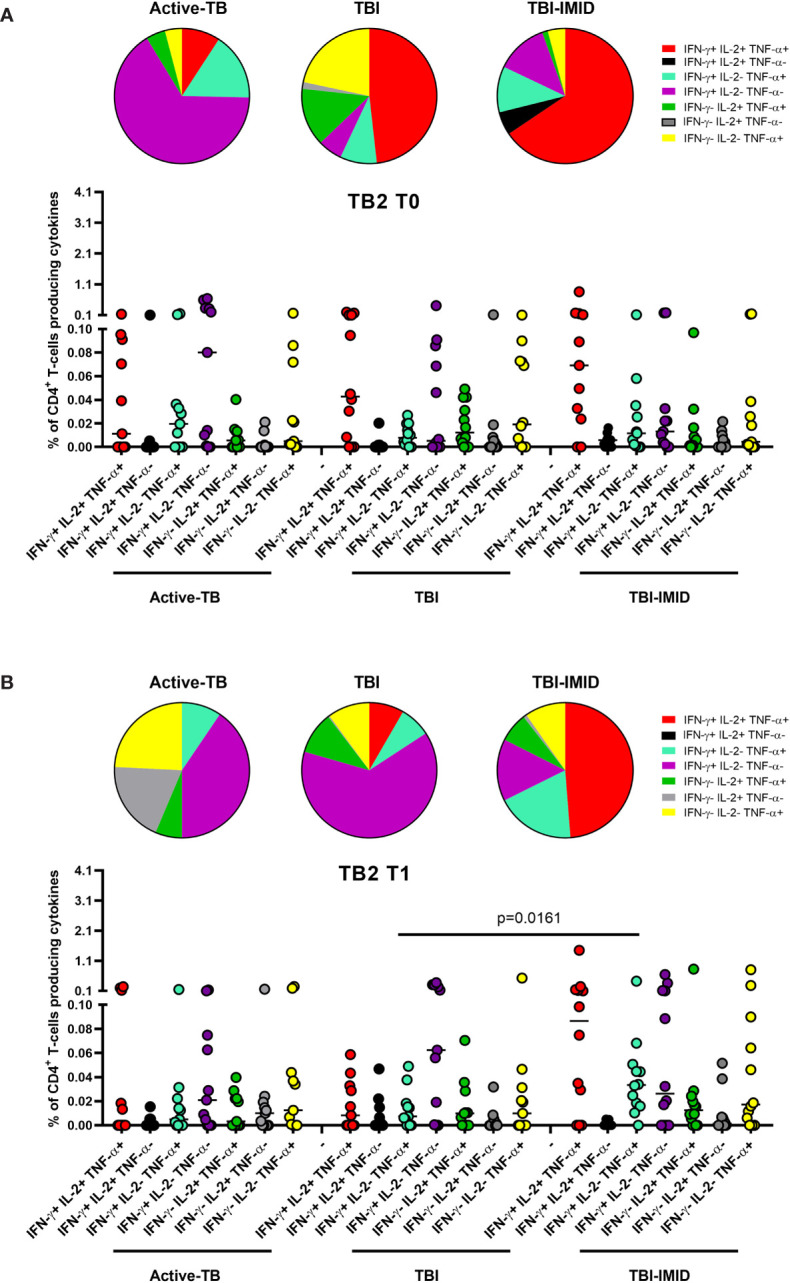
Comparison of functional profile of TB2 specific CD4 T cells at each time point. PBMC of patients enrolled before (T0) and after TB therapy (T1) were stimulated overnight with TB2 peptides. **(A)** T0; **(B)** T1. Cytokine profile was evaluated only on responders using Boolean gate combination. The number of CD4 and CD8 responders to TB1 and TB2 stimulation is reported in detail in **Table 3**. Horizontal lines indicate the median. Statistical analysis was performed using the Mann–Whitney unmatched test, Bonferroni correction was applied, and the p value was considered significant if ≤0.017. TB, tuberculosis; TBI, tuberculosis infection; IMID, immune mediated inflammatory disease; TB1, peptides of TB1 tube of QFT-plus kit; TB2, peptides of TB2 tube of QFT-plus kit.

Then, we compared the cytokine profile of Mtb-specific CD4 T cells among the different groups at each time point (**Figures 6** and **7**). In response to TB1 stimulation at T0, we did not observe any significant differences (**Figure 6A**). However, the main involved subset was constituted by the triple functional IFN-γ^+^ IL-2^+^ TNF-α^+^ CD4^+^ T cells in all groups; in active-TB and TBI-IMID, the contribution of monofunctional IFN-γ^+^ IL-2^-^ TNF-α^-^ CD4^+^ T cells was also evident (**Figure 6A**). Similarly, in response to TB1 stimulation at T1, we did not observe any significant differences (**Figure 6B**). In particular, we observed: in active-TB a high proportion of monofunctional IFN-γ^+/-^ IL-2^-^ TNF-α^+/-^ CD4^+^ T cells and triple functional IFN-γ^+^ IL-2^+^ TNF-α^+^ CD4^+^ T cells; in TBI, a great contribution of monofunctional IFN-γ^+^ IL-2^-^ TNF-α^-^ CD4^+^ T cells; in TBI-IMID, a high proportion of monofunctional IFN-γ^+^ IL-2^-^ TNF-α^-^ CD4^+^ T cells and triple functional IFN-γ^+^ IL-2^+^ TNF-α^+^ CD4^+^ T cells (**Figure 6B**).

In response to TB2 stimulation at T0, we did not observe any significant differences (**Figure 7A**). However, in active-TB, the monofunctional IFN-γ^+^ IL-2^-^ TNF-α^-^ CD4^+^ T cells constituted the main involved subset, whereas in TBI and TBI-IMID the triple functional IFN-γ^+^ IL-2^+^ TNF-α^+^ CD4^+^ T cells was the major represented subset (**Figure 7A**). In response to TB2 stimulation at T1 (**Figure 7B**), we observed a high contribution of the monofunctional IFN-γ^+^ IL-2^-^ TNF-α^-^ CD4^+^ T cells in active-TB and TBI. In TBI-IMID, we reported a high and significant percentage of double functional IFN-γ^+^ IL-2^-^ TNF-α^+^ CD4^+^ T cells compared to TBI (p = 0.0161) and a high contribution of triple functional IFN-γ^+^ IL-2^+^ TNF-α^+^ CD4^+^ T cells in TBI-IMID (**Figure 7B**).

Collectively, these data indicated at T0 a predominant TB1-induced polyfunctional profile and at T1 an increase of TB1-induced monofunctional IFN-γ^+^ IL-2^-^ TNF-α^-^ CD4^+^ T cells in all groups (**Figure 6**). The TB2 cytokine profile was more heterogeneous, showing that triple functional IFN-γ^+^ IL-2^+^ TNF-α^+^ CD4^+^ T cells were mainly represented in TBI-IMID patients at both T0 and T1 (**Figure 7**).

### TB Treatment Did Not Affect the Phenotype of Mtb-Specific T Cells

**Figure 8:**
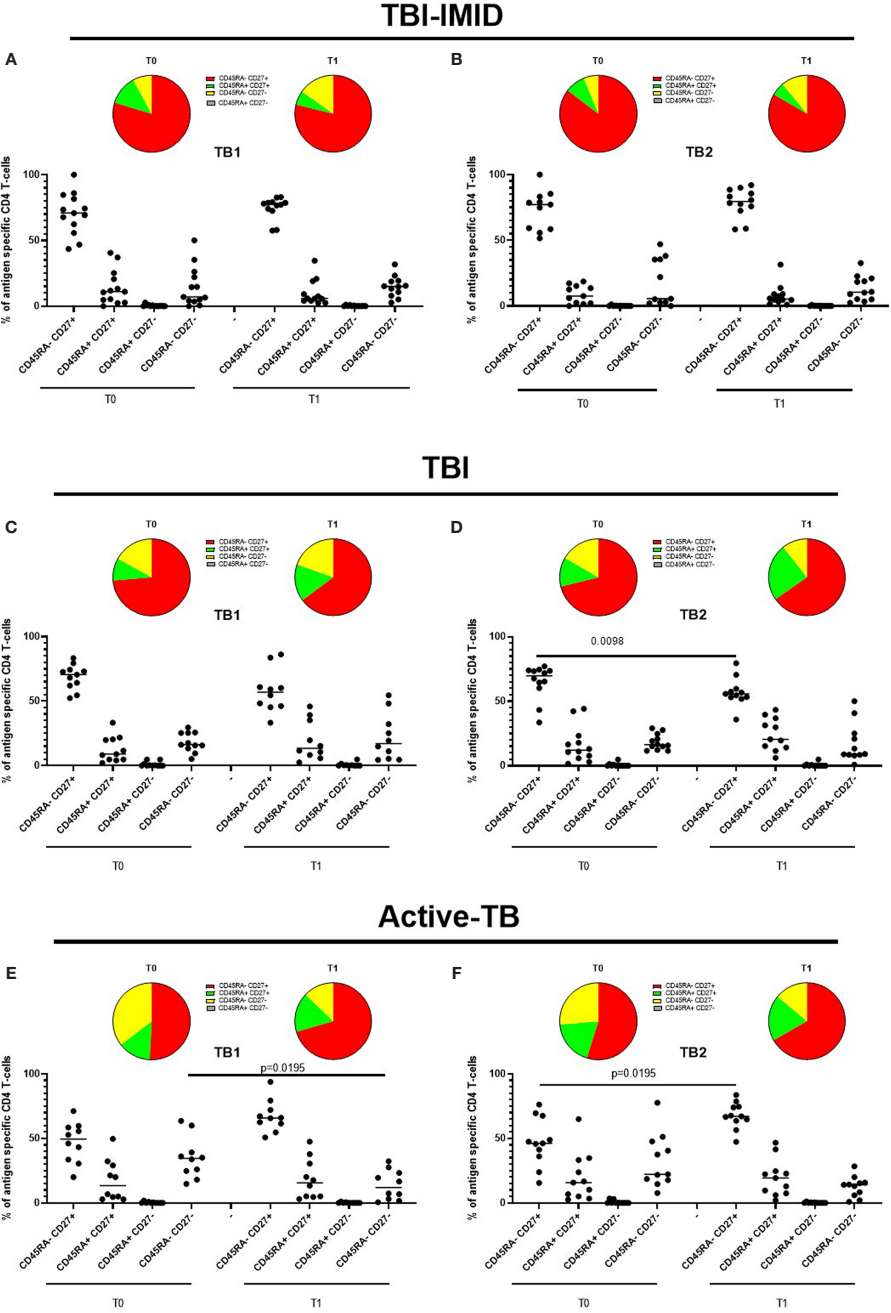
Phenotype of Mtb-specific CD4 T cells before and after TB therapy completion. PBMC of patients enrolled before (T0) and after TB therapy (T1) were stimulated overnight with TB1 and TB2 peptides, and CD45RA and CD27 proportions were evaluated on total cytokine response and T cells producing IFN-γ, IL-2, and TNF-α. **(A, B)** TBI-IMID patients; **(C, D)** TBI subjects; **(E, F)** Active-TB patients. Phenotype was evaluated only on responders. The number of CD4 and CD8 responders to TB1 and TB2 stimulation is reported in detail in **Table 3**. Horizontal lines indicate the median. Statistical analysis was performed using the Wilcoxon matched-pairs signed rank test, and the p value was considered significant if ≤0.05. TB, tuberculosis; TBI, tuberculosis infection; IMID, immune mediated inflammatory disease; TB1, peptides of TB1 tube of QFT-plus kit; TB2, peptides of TB2 tube of QFT-plus kit.

Next, we investigated if the TB treatment had or not an impact on the phenotype of Mtb-specific T cells, evaluating the proportion of CD45RA and CD27 (**Figure 8**). Even in this case, we focused on CD4 T cells since the number of CD8 T cells responders was too low to perform a robust statistical analysis (**Table 3**).

Since we did not observe differences in term of FDS and considering the low frequency of the Mtb-specific T cells, we decided to analyze the phenotype of Mtb-specific CD4 T cells able to produce any cytokines (total response based on IFN-γ, TNF-α, or IL-2 production). In this way, we had the maximum number of events available for the phenotype description, and we avoided as much as possible the interference of the background that could not be subtracted in the phenotype evaluation.

In TBI-IMID and TBI subjects, the Mtb-specific CD4 T-cell response was mainly represented by the CD45RA^-^CD27^+^ subset both at T0 and T1 (with a frequency ranging from 55% to 79%) (**Figures 8A–D**). In TBI-IMID patients, we did not observe significant differences in response to TB1 and TB2 antigens and any evident trend (**Figures 8A, B**). In TBI subjects, we observed a lower proportion of Mtb-specific CD45RA^-^ CD27^+^ CD4 T cells at T1 compared to T0 (p = 0.0098) in response to TB2 and a similar trend in response to TB1 (**Figures 8C, D**).

In active-TB, the Mtb-specific CD4 T-cell response was mainly represented by the CD45RA^-^ CD27^+^ subset both a T0 and T1 (with a frequency ranging from 46% to 67%) (**Figures 8E, F**). We observed an increased Mtb-specific CD4^+^ CD45RA^-^ CD27^+^ subset and a contraction of the CD45RA^-^ CD27^-^ subset after therapy completion in response to TB1 and TB2 antigens (**Figures 8E, F**). The frequency of the CD45RA^-^ CD27^+^ subset was significantly increased in response to TB2 (p = 0.0195, **Figure 8F**), whereas the frequency of the CD45RA^-^ CD27^-^ subset was significantly decreased in response to TB1 (p = 0.0195, **Figure 8E**).

**Figure 9:**
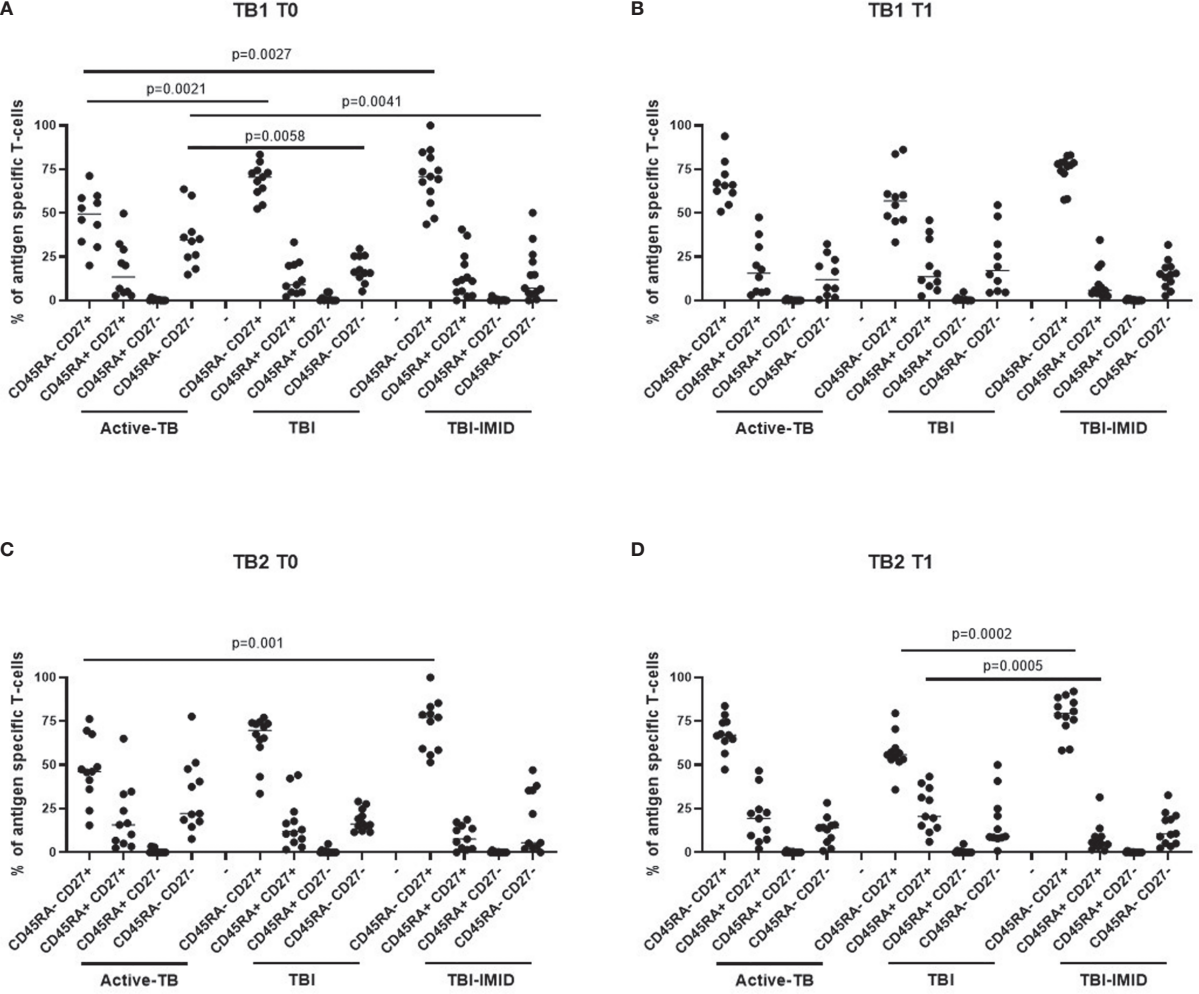
Comparison of phenotype of Mtb-specific CD4 T cells at each time point. PBMC of patients enrolled before (T0) and after TB therapy (T1) were stimulated overnight with TB1 and TB2 peptides, and CD45RA and CD27 proportions were evaluated on total cytokine response and T cells producing IFN-γ, IL-2, and TNF-α. **(A, B)** TB1 stimulation at T0 and T1; **(C, D)** TB2 stimulation at T0 and T1. Phenotype was evaluated only on responders. The number of CD4 and CD8 responders to TB1 and TB2 stimulation is reported in detail in **Table 3**. Horizontal lines indicate the median. Statistical analysis was performed using the Mann–Whitney unmatched test, Bonferroni correction was applied, and the p value was considered significant if ≤0.017. TB, tuberculosis; TBI, tuberculosis infection; IMID, immune mediated inflammatory disease; TB1, peptides of TB1 tube of QFT-plus kit; TB2, peptides of TB2 tube of QFT-plus kit.

Then, we compared the phenotype profile of Mtb-specific CD4 T cells among the different groups at each time point (**Figure 9**). In response to TB1 stimulation at T0, we found that the CD45RA^-^CD27^+^ subset was significantly lower in active-TB compared to TBI and TBI-IMID (p = 0.0021; p = 0.0027) and the CD45RA^-^CD27^-^ subset was significantly higher in active-TB compared to TBI and TBI-IMID (p = 0.0058; p = 0.0041) (**Figure 9A**). Differently, at T1, we did not find any significant differences (**Figure 9B**).

In response to TB2 stimulation at T0, we found a phenotype profile similar to TB1 stimulation for all groups; however, only the CD45RA^-^CD27^+^ subset was significantly lower in active-TB compared to TBI-IMID (p = 0.001) (**Figure 9C**). At T1, we observed that the TBI subjects had lower proportion of the CD45RA^-^CD27^+^ subset (p = 0.0002) and a higher proportion of CD45RA^+^CD27^+^ (p = 0.0005) compared to TBI-IMID (**Figure 9D**).

Collectively, these data highlighted a loss of CD27^+^ CD4 T cells in active-TB patients and this profile was more evident at T0.

## Discussion

Among patients with dysregulation of the immune system, subjects with IMID, such as RA, PsA, and AS, have an intrinsic higher probability to develop active-TB compared to the general population (7, 10). The risk of Mtb reactivation in the general population of TBI subjects usually is the highest during the first 2 years after Mtb exposure (3, 4, 6).

According to the WHO, the prevalence of RA varies between 0.3% and 1% and is more common in women and in developed countries (7–10). Therefore, the identification and treatment of TBI in this fragile population is important to propose the TB preventive therapy (7–10).

In this study, we explored the Mtb-specific immunity of TBI-IMID patients at the baseline and after TB therapy completion. The main finding of this study regards the CD4 T-cell response. We demonstrate in TBI-IMID that TB therapy did not affect the ability of CD4 T cells to produce IFN-γ, TNF-α, and IL-2, their functional status, and their phenotype. Moreover, we demonstrated that at the baseline TBI subjects and TBI-IMID patients had a similar phenotype that instead was different from active-TB patients. We enrolled two different categories of controls: the TBI without IMID to study the Mtb-specific immune response in a population controlling the Mtb replication and the active-TB group as a positive control of the ongoing Mtb replication. TB treatment did not significantly affect the CD4 cytokine production neither in the TBI nor in the active-TB group. As previously demonstrated, the CD8 T-cell response was detected in lower proportion compared to the CD4 T-cell response (48, 50). The CD4 functional status in TBI subjects showed a significant decrease of triple functional T cells after treatment and in active-TB a not significant increase of the proportion of monofunctional IFN-γ^+^ IL-2^-^ TNF-α^-^. The baseline level of polyfunctional IFN-γ^+^ IL-2^+^ TNF-α^+^ CD4 T cells in both TBI-IMID and TBI was similar to the results reported in other TBI population (29, 31, 47).

The comparison among groups at each time point confirmed as shown by the pair-wise comparison that at T0 all the studied groups had a similar cytokine profile mainly constituted by the triple functional IFN-γ^+^ IL-2^+^ TNF-α^+^ CD4 T-cell subset and at T1 all groups had a notable proportion of monofunctional IFN-γ^+^ IL-2^-^ TNF-α^-^ CD4 T cells.

Since it has been demonstrated that recent and remote TBI subjects, naïve for TB therapy, had a similar CD4 cytokine profile (29), the functional differences after TB therapy between TBI and TBI-IMID individuals were not ascribable to their remote or recent Mtb exposure but probably to the IMID status itself. TB therapy did not affect neither the functional differentiation status of CD4 T cells in the TBI, TBI-IMID, and active-TB groups. Considering that the majority of TBI had a recent infection and the majority of TBI-IMID had a remote exposure, our data were in line with recent findings on recent subsets and persistent QFT^+^ individuals (29).

In active-TB, the baseline functional status of TB1 CD4-specific T cells was in agreement with previous reports reporting a high proportion of the IFN-γ^+^ IL-2^+^ TNF-α^+^ CD4 T-cell subset in response to ESAT-6 CFP-10 stimulation (25, 31, 54). For the TB2 stimulation, the functional status skewed towards the IFN-γ^+^ IL-2^-^ TNF-α^-^ CD4 T cells, maybe as a consequence of the ability of TB2 peptides to stimulate also the CD8 T cells (48).

T-cell expression of surface molecules such as CD45RA, CD27, and CCR7 identifies different T-cell subsets that reflect different stages of cell differentiation (55). The effector T cells are expanded during active-Mtb replication, whereas the memory cells associate with control and eradication of Mtb-infection (31, 47, 56). The phenotype characterization demonstrated a great contribution of the CD45RA^-^ CD27^+^ subset to the Mtb-specific immune response in all groups. The presence of CD27 could be associated to both central memory (CD45RA^-^ CD27^+^ CCR7^+^) and effector memory subsets (CD45RA^-^ CD27^+^ CCR7^-^) (29, 55). Since we did not evaluate the CCR7 expression, we could not assign a precise memory status to the different subsets evaluated. However, as previously demonstrated (31), at the baseline, the Mtb-specific CD45RA^-^ CD27^+^ CD4 T cells were more represented in the TBI individuals, whereas the active-TB patients had a higher proportion of Mtb-specific CD45RA^-^ CD27^-^ CD4 T cells compared to TBI subjects. These findings reflect the loss of CD27 during active-TB disease, already documented in several studies (38–41, 57). Interestingly, after therapy completion, the active-TB patients had a phenotype more similar to TBI groups, with an increased proportion of the CD45RA^-^ CD27^+^ subset and a contraction of the CD45RA^-^ CD27^-^ CD4 T cells. These data suggest that the high Mtb load in active-TB patients induces the differentiation of Mtb-specific CD45RA^-^ CD27^-^ CD4 T cells, whereas the low Mtb load of TBI individuals favours the CD45RA^-^ CD27^+^ subset.

The phenotype comparison among groups at each time point confirmed previous study on CD27 expression in patients with different TB status (31, 38, 40). Indeed, at the baseline, the TBI subjects had a higher proportion of Mtb-specific CD45RA^-^ CD27^+^ CD4 T cells compared to active-TB and the active-TB a higher proportion of Mtb-specific CD45RA^-^ CD27^-^ CD4 T cells compared to TBI subjects. These data reflected the modulation of CD27 according to the Mtb bacterial load (39, 41). Interestingly, the TBI-IMID patients showed a phenotype profile similar to TBI subjects, suggesting that the type of IMID and the concomitant IMID therapy did not affect the activation status of Mtb-specific CD4 T cells. After TB therapy completion, the differences among Mtb-specific CD45RA^-^ CD27^+^ CD4 T cells and CD45RA^-^ CD27^-^ CD4 T cells were less evident, suggesting that TB therapy, decreasing the Mtb load, led to an increased CD27 expression.

Moreover, lately, recent and remote TBI have been shown to have a similar phenotype according to the expression of CD45RA CD27 CCR7 markers (29). Therefore, the phenotype of TBI-IMID individuals was not even due to their remote Mtb exposure. Larger studies are needed to better understand the modulation of the phenotype in these IMID patients.

Considering that the IMID therapies were mostly based on the modulation of the immune system, indirectly we could suppose that the type of IMID therapy did not affect the immune response. Stratifying for the presence or not of biologic drugs, we did not find any significant differences among TBI-IMID before and after therapy completion. Although the sample size did not permit a deeper analysis, in support of these findings, we have previously demonstrated that neither the number of lymphocytes nor the type of IMID therapy influenced the IFN-γ response to QFT-Plus in TBI-IMID patients (28). Due to the adult age of the IMID manifestations, the TBI-IMID were older compared to TBI subjects. As lymphocyte counts decline with age (58) and potentially contribute to the immune impairment, we evaluated the lymphocyte counts in this study. No significant differences were found nor comparing all the three groups at each time points, neither comparing the two time points within each group. An exception was found considering those with active-TB in which a restore of the lymphocyte counts was observed after therapy completion (59). Moreover, the similar cytokine profile and phenotype of TBI subjects and TBI-IMID patients seem to support the presence of a comparable immune response to Mtb antigens. Therefore, as the immunity status at the different time points was comparable, the data reported could be considered reliable. Although these data are preliminary, we highlighted differences between the CD4 T-cell cytokine response over time of TBI-IMID compared to TBI and active-TB. Surprisingly, the monofunctional IFN-γ^+^ IL-2^-^ TNF-α^-^ CD4 T cells characterized the TBI and active-TB after therapy completion, whereas the TBI-IMID patients maintain a cytokine profile similar to the baseline. It is not possible to state if TB therapy induced a protective cytokine profile in TBI-IMID; however, these patients have been followed for 6 years and none of them developed until now active-TB disease. Since the phenotype studied based on CD45RA and CD27 did not allow to define a particular profile over time, we may need to better characterize the response using also other activation markers such as HLA-DR (29, 60).

Limitations of this longitudinally study are the relatively low number of patients enrolled, the variety of the IMID considered, and the different regimens of IMID therapy. However, since only few studies are available on Mtb immune response in this fragile population of TBI subjects, we retain this is a good controlled study including two types of control populations (TBI and active-TB). More importantly, besides the reported caveats, we answered to opened important questions on Mtb-specific response in this particular understudied category of fragile TBI subjects at high risk to progress to active-TB disease.

In conclusions, we evaluated over time the modulation of Mtb-specific immune response in patients at different stages of TB: a low-replicating Mtb status in TBI, a low-replicating Mtb status in TBI-IMID patients with high risk to develop the active-TB disease, and a high-replicating Mtb status in active-TB patients. The TB therapy did not modify the CD4 T-cell cytokine profile of TBI-IMID patients but determined a contraction of the triple functional CD4 T cells of the TBI subjects and active-TB patients. The CD45RA^-^ CD27^+^ T cells stood out as a main subset of the Mtb-specific response in all groups of patients. Before the TB-preventive therapy, the TBI subjects had higher proportion of Mtb-specific CD45RA^-^CD27^+^CD4^+^ T cells and the active-TB higher proportion of Mtb-specific CD45RA^-^CD27^-^CD4^+^ T cells compared to other groups. The TBI-IMID patients showed a phenotype similar to TBI, suggesting that the type of IMID and the IMID therapy did not affect the activation status of Mtb-specific CD4 T cells.

Future studies on a larger and better-stratified TBI-IMID population will help to understand the change of the Mtb-specific immune response over time and to identify possible immune biomarkers of Mtb-containment or active replication.

## Data Availability Statement

The datasets presented in this study can be found in online repositories. The names of the repository/repositories and accession number(s) can be found below: The data sets generated during and/or analyzed during the current study are available in our institutional repository after request at rawdata.inmi.it.

## Ethics Statement

The study involving human participants were approved by the Ethical Committee of “L. Spallanzani” National Institute of Infectious Diseases (INMI)-IRCCS, approval number 72/2015. The study was performed following the guidelines of the Declaration of Helsinki. The patients/participants provided their written informed consent to participate in this study.

## Author Contributions

EP performed the experiments, analyzed and interpreted data, and wrote the manuscript. LP analyzed and interpreted data and revised the manuscript. TC performed the experiments. CF analyzed and interpreted data and revised the manuscript. GC enrolled patients and collected clinical data. AN performed the statistical analysis and interpreted data. VV processed blood samples. UM enrolled patients and revised the manuscript. ML, GG, NC, and FC revised the manuscript and participated in the interpretation of data. FP enrolled patients and revised the manuscript. DG designed and wrote the study, coordinated and supervised the project, contributed to the interpretation of the results, and revised the manuscript. All authors contributed to the article and approved the submitted version.

## Funding

This study was funded by GR-2018-12367178 and Ricerca Corrente by Line four, all funded by Italian Ministry of Health.

## Conflict of Interest

The authors declare that the research was conducted in the absence of any commercial or financial relationships that could be construed as a potential conflict of interest.

## Publisher’s Note

All claims expressed in this article are solely those of the authors and do not necessarily represent those of their affiliated organizations, or those of the publisher, the editors and the reviewers. Any product that may be evaluated in this article, or claim that may be made by its manufacturer, is not guaranteed or endorsed by the publisher.

## Supplementary Material

Supplementary Figure 1 IFN-γ response to antigens present in QFT-Plus test, TB1 and TB2, before and at the end of TB treatment. **(A, B)** TBI-IMID patients; **(C, D)** TBI subjects; **(E, F)** Active-TB patients. Statistical analysis was performed using the Wilcoxon matched-pairs signed rank test, and the p value was considered significant if ≤0.05. Two TB-IMID and one active-TB patients did not perform the test at T1. Number of responders over total enrolled patients is reported below each panel. TB, tuberculosis; TBI, tuberculosis infection; IMID, immune mediated inflammatory disease; TB1, peptides of TB1 tube of QFT-plus kit; TB2, peptides of TB2 tube of QFT-plus kit; IU/ml, international unit at millilitre; T0, before TB therapy, T1, after TB therapy completion.

Supplementary Figure 2 Cytokine production CD4 and CD8 T cells in response to mitogen stimulation before and after TB therapy completion: PBMC of patients enrolled before (T0) and after TB therapy (T1) were stimulated overnight with Mitogen reagent of QFT-Plus kit. **(A, B)** TBI-IMID patients; **(C, D)** TBI subjects; **(E, F)** Active-TB patients. Number of responders over total enrolled patients is reported below each graph. Horizontal lines indicate the median. Statistical analysis was performed using the Wilcoxon matched-pairs signed rank test, and the p value was considered significant if ≤0.05 for cytokine production. TB, tuberculosis; TBI, tuberculosis infection; IMID, immune mediated inflammatory disease.

Supplementary Figure 3 Comparison of cytokine production CD4 and CD8 T cells in response to mitogen stimulation: PBMC of patients enrolled before (T0) and after TB therapy (T1) were stimulated overnight with Mitogen reagent of QFT-Plus kit. **(A)** T0; **(B)** T1. Number of responders over total enrolled patients is reported below each graph. Horizontal lines indicate the median. Statistical analysis was performed using the Mann–Whitney unmatched test, Bonferroni correction was applied, and the p value was considered significant if ≤0.017 for cytokine production. TB, tuberculosis; TBI, tuberculosis infection; IMID, immune mediated inflammatory disease.

Supplementary Figure 4 Functional differentiation score (FDS) of Mtb-specific CD4 T cells is similar before and after TB therapy in TBI-IMID, TBI subjects, and active-TB patients. FDS has been calculated as previously described (28, 29) only on CD4 T-cell responders to Mtb antigen stimulation. **(A, B)** TBI-IMID patients; **(C, D)** TBI subjects; **(E, F)** Active-TB patients. Horizontal lines indicate the median. The number of CD4 and CD8 responders to TB1 and TB2 stimulation is reported in detail in **Table 3**. Statistical analysis was performed using the Wilcoxon matched-pairs signed rank test, and the p value was considered significant if ≤0.05. TB, tuberculosis; TBI, tuberculosis infection; IMID, immune mediated inflammatory disease; TB1, peptides of TB1 tube of QFT-plus kit; TB2, peptides of TB2 tube of QFT-plus kit; T0, before TB therapy; T1, after TB therapy completion.

Supplementary Figure 5 Functional differentiation score (FDS) of Mtb-specific CD4 T cells is similar before and after TB therapy in TBI-IMID remote and TBI recent subjects. FDS has been calculated as previously described (28, 29) only on CD4 T-cell responders to Mtb antigen stimulation. **(A, B)** TBI-IMID remote patients, n = 12; **(C, D)** TBI recent subjects, N = 8. Horizontal lines indicate the median. Statistical analysis was performed using the Wilcoxon matched-pairs signed rank test, and the p value was considered significant if ≤0.05. TB, tuberculosis; TBI, tuberculosis infection; IMID, immune mediated inflammatory disease; TB1, peptides of TB1 tube of QFT-plus kit; TB2, peptides of TB2 tube of QFT-plus kit; T0, before TB therapy; T1, after TB therapy completion.

## References

[1] WHO. TB REPORT 2020. (2020). Available at: https://www.who.int/publications/i/item/9789240013131.

[2] Houben RM Dodd PJ. The Global Burden of Latent Tuberculosis Infection: A Re-Estimation Using Mathematical Modelling. PloS Med (2016) 13:e1002152. 10.1371/journal.pmed.1002152 27780211 PMC5079585

[3] Goletti D Lee MR Wang JY Walter N Ottenhoff THM. Update on Tuberculosis Biomarkers: From Correlates of Risk, to Correlates of Active Disease and of Cure From Disease. Respirology (2018) 23:455–66. 10.1111/resp.13272 29457312

[4] Behr MA Edelstein PH Ramakrishnan L. Revisiting the Timetable of Tuberculosis. BMJ (2018) 362:k2738. 10.1136/bmj.k2738 30139910 PMC6105930

[5] Petruccioli E Scriba TJ Petrone L Hatherill M Cirillo DM Joosten SA. Correlates of Tuberculosis Risk: Predictive Biomarkers for Progression to Active Tuberculosis. Eur Respir J (2016) 48:1751–63. 10.1183/13993003.01012-2016 PMC5898936 27836953

[6] Trauer JM Moyo N Tay EL Dale K Ragonnet R McBryde ES. Risk of Active Tuberculosis in the Five Years Following Infection . . 15%? Chest (2016) 149:516–25. 10.1016/j.chest.2015.11.017 26867835

[7] Cantini F Nannini C Niccoli L Iannone F Delogu G Garlaschi G. Guidance for the Management of Patients With Latent Tuberculosis Infection Requiring Biologic Therapy in Rheumatology and Dermatology Clinical Practice. Autoimmun Rev (2015) 14:503–9. 10.1016/j.autrev.2015.01.011 25617816

[8] Cantini F Nannini C Niccoli L Petrone L Ippolito G Goletti D. Risk of Tuberculosis Reactivation in Patients With Rheumatoid Arthritis, Ankylosing Spondylitis, and Psoriatic Arthritis Receiving Non-Anti-TNF-Targeted Biologics. Mediators Inflamm (2017) 2017:8909834. 10.1155/2017/8909834 28659665 PMC5474286

[9] Cantini F Niccoli L Goletti D. Tuberculosis Risk in Patients Treated With Non-Anti-Tumor Necrosis Factor-α (TNF-α) Targeted Biologics and Recently Licensed TNF-α Inhibitors: Data From Clinical Trials and National Registries. J Rheumatol Suppl (2014) 91:56–64. 10.3899/jrheum.140103 24789001

[10] Goletti D Petrone L Ippolito G Niccoli L Nannini C Cantini F. Preventive Therapy for Tuberculosis in Rheumatological Patients Undergoing Therapy With Biological Drugs. Expert Rev Anti Infect Ther (2018) 16:501–12. 10.1080/14787210.2018.1483238 29848120

[11] Cantini F Niccoli L Capone A Petrone L Goletti D. Risk of Tuberculosis Reactivation Associated With Traditional Disease Modifying Anti-Rheumatic Drugs and Non-Anti-Tumor Necrosis Factor Biologics in Patients With Rheumatic Disorders and Suggestion for Clinical Practice. Expert Opin Drug Saf (2019) 18:415–25. 10.1080/14740338.2019.1612872 31066297

[12] Nash P Kerschbaumer A Dörner T Dougados M Fleischmann RM Geissler K. Points to Consider for the Treatment of Immune-Mediated Inflammatory Diseases With Janus Kinase Inhibitors: A Consensus Statement. Ann Rheum Dis (2021) 80:71–87. 10.1136/annrheumdis-2020-218398 33158881 PMC7788060

[13] Cantini F Blandizzi C Niccoli L Petrone L Goletti D. Systematic Review on Tuberculosis Risk in Patients With Rheumatoid Arthritis Receiving Inhibitors of Janus Kinases. Expert Opin Drug Saf (2020) 19:861–72. 10.1080/14740338.2020.1774550 32552289

[14] Saliu OY Sofer C Stein DS Schwander SK Wallis RS. Tumor-Necrosis-Factor Blockers: Differential Effects on Mycobacterial Immunity. J Infect Dis (2006) 194:486–92. 10.1086/505430 16845632

[15] Goletti D Carrara S Butera O Amicosante M Ernst M Sauzullo I. Accuracy of Immunodiagnostic Tests for Active Tuberculosis Using Single and Combined Results: A Multicenter TBNET-Study. PloS One (2008) 3:e3417. 10.1371/journal.pone.0003417 18923709 PMC2561073

[16] Goletti D Carrara S Vincenti D Saltini C Rizzi EB Schininà V. Accuracy of an Immune Diagnostic Assay Based on RD1 Selected Epitopes for Active Tuberculosis in a Clinical Setting: A Pilot Study. Clin Microbiol Infect (2006) 12:544–50. 10.1111/j.1469-0691.2006.01391.x 16700703

[17] Goletti D Navarra A Petruccioli E Cimaglia C Compagno M Cuzzi G. Latent Tuberculosis Infection Screening in Persons Newly-Diagnosed With HIV Infection in Italy: A Multicentre Study Promoted by the Italian Society of Infectious and Tropical Diseases. Int J Infect Dis (2020) 92:62–8. 10.1016/j.ijid.2019.12.031 31887456

[18] Kik SV Schumacher S Cirillo DM Churchyard G Boehme C Goletti D. An Evaluation Framework for New Tests That Predict Progression From Tuberculosis Infection to Clinical Disease. Eur Respir J (2018) 52:1800946. 10.1183/13993003.00946-2018 30139776

[19] Abubakar I Drobniewski F Southern J Sitch AJ Jackson C Lipman M. Prognostic Value of Interferon-γ Release Assays and Tuberculin Skin Test in Predicting the Development of Active Tuberculosis (UK PREDICT TB): A Prospective Cohort Study. Lancet Infect Dis (2018) 18:1077–87. 10.1016/S1473-3099(18)30355-4 PMC6192014 30174209

[20] Wong SH Gao Q Tsoi KK Wu WK Tam LS Lee N. Effect of Immunosuppressive Therapy on Interferon γ Release Assay for Latent Tuberculosis Screening in Patients With Autoimmune Diseases: A Systematic Review and Meta-Analysis. Thorax (2016) 71:64–72. 10.1136/thoraxjnl-2015-207811 26659461

[21] Igari H Ishikawa S Nakazawa T Oya Y Futami H Tsuyuzaki M. Lymphocyte Subset Analysis in QuantiFERON-TB Gold Plus and T-Spot.TB for Latent Tuberculosis Infection in Rheumatoid Arthritis. J Infect Chemother (2018) 24:110–6. 10.1016/j.jiac.2017.09.012 29054459

[22] Nemes E Rozot V Geldenhuys H Bilek N Mabwe S Abrahams D. Optimization and Interpretation of Serial QuantiFERON Testing to Measure Acquisition of Mycobacterium Tuberculosis Infection. Am J Respir Crit Care Med (2017) 196:638–48. 10.1164/rccm.201704-0817OC PMC5620669 28737960

[23] Chiacchio T Petruccioli E Vanini V Cuzzi G La Manna MP Orlando V. Impact of Antiretroviral and Tuberculosis Therapies on CD4(+) and CD8(+) HIV/M. Tuberculosis-Specific T-Cell in Co-Infected Subjects. Immunol Lett (2018) 198:33–43. 10.1016/j.imlet.2018.04.001 29635002

[24] Andrews JR Noubary F Walensky RP Cerda R Losina E Horsburgh CR. Risk of Progression to Active Tuberculosis Following Reinfection With Mycobacterium Tuberculosis. Clin Infect Dis (2012) 54:784–91. 10.1093/cid/cir951 PMC3284215 22267721

[25] Day CL Abrahams DA Lerumo L Janse van Rensburg E Stone L O’rie T. Functional Capacity of Mycobacterium Tuberculosis-Specific T Cell Responses in Humans Is Associated With Mycobacterial Load. J Immunol (2011) 187:2222–32. 10.4049/jimmunol.1101122 PMC3159795 21775682

[26] Goletti D Parracino MP Butera O Bizzoni F Casetti R Dainotto D. Isoniazid Prophylaxis Differently Modulates T-Cell Responses to RD1-Epitopes in Contacts Recently Exposed to Mycobacterium Tuberculosis: A Pilot Study. Respir Res (2007) 8(1):5. 10.1186/1465-9921-8-5 17257436 PMC1794408

[27] Petruccioli E Chiacchio T Vanini V Cuzzi G Codecasa LR Ferrarese M. Effect of Therapy on Quantiferon-Plus Response in Patients With Active and Latent Tuberculosis Infection. Sci Rep (2018) 8:15626. 10.1038/s41598-018-33825-w 30353115 PMC6199315

[28] Chiacchio T Petruccioli E Vanini V Cuzzi G Massafra U Baldi G. Characterization of QuantiFERON-TB-Plus Results in Latent Tuberculosis Infected Patients With or Without Immune-Mediated Inflammatory Diseases. J Infect (2019) 79:15–23. 10.1016/j.jinf.2019.04.010 30981891

[29] Mpande CAM Rozot V Mosito B Musvosvi M Dintwe OB Bilek N. Immune Profiling of Mycobacterium Tuberculosis-Specific T Cells in Recent and Remote Infection. EBioMedicine (2021) 64:103233. 10.1016/j.ebiom.2021.103233 33610126 PMC7902886

[30] Moguche AO Musvosvi M Penn-Nicholson A Plumlee CR Mearns H Geldenhuys H. Antigen Availability Shapes T Cell Differentiation and Function During Tuberculosis. Cell Host Microbe (2017) 21:695–706.e5. 10.1016/j.chom.2017.05.012 28618268 PMC5533182

[31] Petruccioli E Petrone L Vanini V Sampaolesi A Gualano G Girardi E. Ifnγ/Tnfα Specific-Cells and Effector Memory Phenotype Associate With Active Tuberculosis. J Infect (2013) 66:475–86. 10.1016/j.jinf.2013.02.004 23462597

[32] Prezzemolo T Guggino G La Manna MP Di Liberto D Dieli F Caccamo N. Functional Signatures of Human CD4 and CD8 T Cell Responses to Mycobacterium Tuberculosis. Front Immunol (2014) 5:180. 10.3389/fimmu.2014.00180 24795723 PMC4001014

[33] Sallin MA Kauffman KD Riou C Du Bruyn E Foreman TW Sakai S. Host Resistance to Pulmonary Mycobacterium Tuberculosis Infection Requires CD153 Expression. Nat Microbiol (2018) 3:1198–205. 10.1038/s41564-018-0231-6 30202016

[34] Du Bruyn E Ruzive S Lindestam Arlehamn CS Sette A Sher A Barber DL. Mycobacterium Tuberculosis-Specific CD4 T Cells Expressing CD153 Inversely Associate With Bacterial Load and Disease Severity in Human Tuberculosis. Mucosal Immunol (2021) 14:491–9. 10.1038/s41385-020-0322-6 PMC7855386 32678272

[35] Adekambi T Ibegbu CC Cagle S Kalokhe AS Wang YF Hu Y. Biomarkers on Patient T Cells Diagnose Active Tuberculosis and Monitor Treatment Response. J Clin Invest (2015) 125:1827–38. 10.1172/JCI77990 PMC4598074 25822019

[36] Riou C Du Bruyn E Ruzive S Goliath RT Lindestam Arlehamn CS Sette A. Disease Extent and Anti-Tubercular Treatment Response Correlates With Mycobacterium Tuberculosis-Specific CD4 T-Cell Phenotype Regardless of HIV-1 Status. Clin Transl Immunol (2020) 9:e1176. 10.1002/cti2.1176 PMC7520805 33005414

[37] Musvosvi M Duffy D Filander E Africa H Mabwe S Jaxa L. T-Cell Biomarkers for Diagnosis of Tuberculosis: Candidate Evaluation by a Simple Whole Blood Assay for Clinical Translation. Eur Respir J (2018) 51:1800153. 10.1183/13993003.00153-2018 29567725

[38] Petruccioli E Navarra A Petrone L Vanini V Cuzzi G Gualano G. Use of Several Immunological Markers to Model the Probability of Active Tuberculosis. Diagn Microbiol Infect Dis (2016) 86:169–71. 10.1016/j.diagmicrobio.2016.06.007 27431433

[39] Nikitina IY Kondratuk NA Kosmiadi GA Amansahedov RB Vasilyeva IA Ganusov VV. Mtb-Specific CD27low CD4 T Cells as Markers of Lung Tissue Destruction During Pulmonary Tuberculosis in Humans. PloS One (2012) 7:e43733. 10.1371/journal.pone.0043733 22937086 PMC3427145

[40] Petruccioli E Petrone L Vanini V Cuzzi G Navarra A Gualano G. Assessment of CD27 Expression as a Tool for Active and Latent Tuberculosis Diagnosis. J Infect (2015) 71:526–33. 10.1016/j.jinf.2015.07.009 26253021

[41] Portevin D Moukambi F Clowes P Bauer A Chachage M Ntinginya NE. Assessment of the Novel T-Cell Activation Marker-Tuberculosis Assay for Diagnosis of Active Tuberculosis in Children: A Prospective Proof-of-Concept Study. Lancet Infect Dis (2014) 14:931–8. 10.1016/S1473-3099(14)70884-9 25185458

[42] Lyadova I Nikitina I. Cell Differentiation Degree as a Factor Determining the Role for Different T-Helper Populations in Tuberculosis Protection. Front Immunol (2019) 10:972. 10.3389/fimmu.2019.00972 31134070 PMC6517507

[43] Prezzemolo T van Meijgaarden KE Franken KLMC Caccamo N Dieli F Ottenhoff THM. Detailed Characterization of Human Mycobacterium Tuberculosis Specific HLA-E Restricted CD8(+) T Cells. Eur J Immunol (2018) 48:293–305. 10.1002/eji.201747184 29124751 PMC6266868

[44] Caccamo N Pietra G Sullivan LC Brooks AG Prezzemolo T La Manna MP. Human CD8 T Lymphocytes Recognize Mycobacterium Tuberculosis Antigens Presented by HLA-E During Active Tuberculosis and Express Type 2 Cytokines. Eur J Immunol (2015) 45:1069–81. 10.1002/eji.201445193 25631937

[45] La Manna MP Orlando V Prezzemolo T Di Carlo P Cascio A Delogu G. HLA-E-Restricted CD8(+) T Lymphocytes Efficiently Control Mycobacterium Tuberculosis and HIV-1 Coinfection. Am J Respir Cell Mol Biol (2020) 62:430–9. 10.1165/rcmb.2019-0261OC 31697586

[46] Riou C Tanko RF Soares AP Masson L Werner L Garrett NJ. Restoration of CD4+ Responses to Copathogens in HIV-Infected Individuals on Antiretroviral Therapy Is Dependent on T Cell Memory Phenotype. J Immunol (2015) 195:2273–81. 10.4049/jimmunol.1500803 PMC4546876 26195814

[47] Chiacchio T Petruccioli E Vanini V Cuzzi G Pinnetti C Sampaolesi A. Polyfunctional T-Cells and Effector Memory Phenotype Are Associated With Active TB in HIV-Infected Patients. J Infect (2014) 69:533–45. 10.1016/j.jinf.2014.06.009 24975174

[48] Petruccioli E Chiacchio T Pepponi I Vanini V Urso R Cuzzi G. First Characterization of the CD4 and CD8 T-Cell Responses to QuantiFERON-TB Plus. J Infect (2016) 73:588–97. 10.1016/j.jinf.2016.09.008 27717779

[49] Petruccioli E Vanini V Chiacchio T Cuzzi G Cirillo DM Palmieri F. Analytical Evaluation of QuantiFERON- Plus and QuantiFERON- Gold In-Tube Assays in Subjects With or Without Tuberculosis. Tuberculosis (Edinb) (2017) 106:38–43. 10.1016/j.tube.2017.06.002 28802403

[50] Rozot V Vigano S Mazza-Stalder J Idrizi E Day CL Perreau M. Mycobacterium Tuberculosis-Specific CD8+ T Cells Are Functionally and Phenotypically Different Between Latent Infection and Active Disease. Eur J Immunol (2013) 43:1568–77. 10.1002/eji.201243262 PMC6535091 23456989

[51] Sauzullo I Scrivo R Mengoni F Ermocida A Coppola M Valesini G. Multi-Functional Flow Cytometry Analysis of CD4+ T Cells as an Immune Biomarker for Latent Tuberculosis Status in Patients Treated With Tumour Necrosis Factor (TNF) Antagonists. Clin Exp Immunol (2014) 176:410–7. 10.1111/cei.12290 PMC4008986 24528189

[52] Savolainen LE Kantele A Knuuttila A Pusa L Karttunen R Valleala H. Combined Expression of IFN-γ, IL-17, and IL-4 mRNA by Recall PBMCs Moderately Discriminates Active Tuberculosis From Latent Mycobacterium Tuberculosis Infection in Patients With Miscellaneous Inflammatory Underlying Conditions. Front Immunol (2016) 7:239. 10.3389/fimmu.2016.00239 27379100 PMC4905973

[53] QIAGEN. What Is QuantiFERON-TB Gold Plus? In: QTF-Plus. (2021). Available at: https://www.quantiferon.com/products/quantiferon-tb-gold-plus-qft-plus/.

[54] Riou C Berkowitz N Goliath R Burgers WA Wilkinson RJ. Analysis of the Phenotype of Mycobacterium Tuberculosis-Specific CD4+ T Cells to Discriminate Latent From Active Tuberculosis in HIV-Uninfected and HIV-Infected Individuals. Front Immunol (2017) 8:968. 10.3389/fimmu.2017.00968 28848561 PMC5554366

[55] Caccamo N Joosten SA Ottenhoff THM Dieli F. Atypical Human Effector/Memory CD4(+) T Cells With a Naive-Like Phenotype. Front Immunol (2018) 9:2832. 10.3389/fimmu.2018.02832 30559746 PMC6287111

[56] Wang X Cao Z Jiang J Niu H Dong M Tong A. Association of Mycobacterial Antigen-Specific CD4(+) Memory T Cell Subsets With Outcome of Pulmonary Tuberculosis. J Infect (2010) 60:133–9. 10.1016/j.jinf.2009.10.048 19878691

[57] Geldmacher C Ngwenyama N Schuetz A Petrovas C Reither K Heeregrave EJ. Preferential Infection and Depletion of Mycobacterium Tuberculosis-Specific CD4 T Cells After HIV-1 Infection. J Exp Med (2010) 207:2869–81. 10.1084/jem.20100090 PMC3005236 21115690

[58] Sansoni P Cossarizza A Brianti V Fagnoni F Snelli G Monti D. Lymphocyte Subsets and Natural Killer Cell Activity in Healthy Old People and Centenarians. Blood (1993) 82:2767–73. 10.1182/blood.V82.9.2767.2767 8219229

[59] La Manna MP Orlando V Dieli F Di Carlo P Cascio A Cuzzi G. Quantitative and Qualitative Profiles of Circulating Monocytes may Help Identifying Tuberculosis Infection and Disease Stages. PloS One (2017) 12:e0171358. 10.1371/journal.pone.0171358 28208160 PMC5313257

[60] Mpande CAM Musvosvi M Rozot V Mosito B Reid TD Schreuder C. Antigen-Specific T Cell Activation Distinguishes Between Recent and Remote Tuberculosis Infection. Am J Respir Crit Care Med (2021) 203:1556–65. 10.1164/rccm.202007-2686OC PMC8483229 33406011

[61] wma. Declaration of Helsinki Ethical Principles for Medical Research Involving Human Subjects. Available at: https://www.wma.net/policies-post/wma-declaration-of-helsinki-ethical-principles-for-medical-research-involving-human-subjects. 19886379

