# Supplementary figures and images for: Mycobacterium tuberculosis Immune Response in Patients With Immune-Mediated Inflammatory Disease
Source: Front Immunol. 2021 Aug 10;12:716857. doi: 10.3389/fimmu.2021.716857 (PMC8382688; doi:10.3389/fimmu.2021.716857)

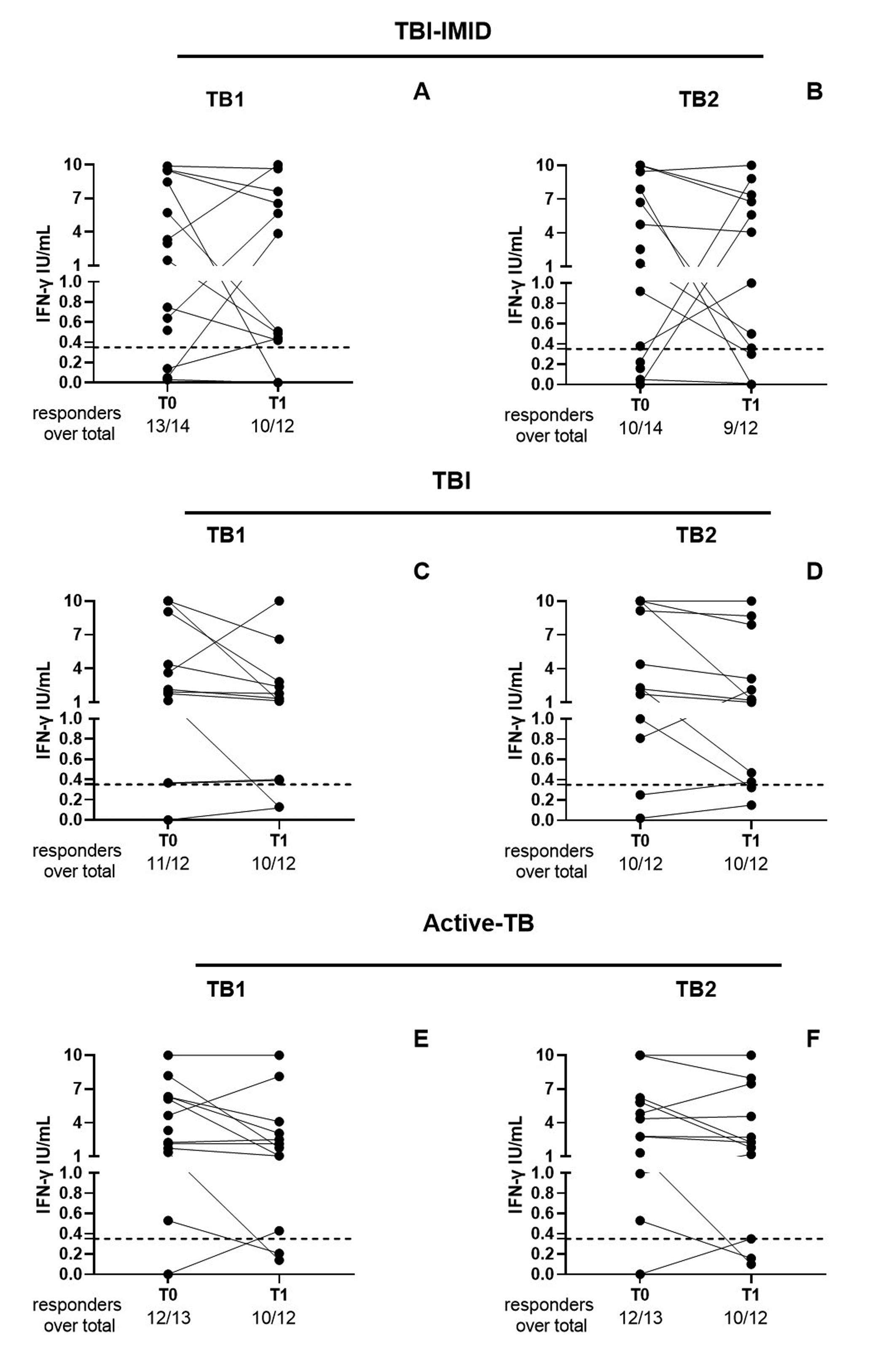

Supplement: Supplementary Figure 1 — IFN-γ response to antigens present in QFT-Plus test, TB1 and TB2, before and at the end of TB treatment. (A, B) TBI-IMID patients; (C, D) TBI subjects; (E, F) Active-TB patients. Statistical analysis was performed using the Wilcoxon matched-pairs signed rank test, and the p value was considered significant if ≤0.05. Two TB-IMID and one active-TB patients did not perform the test at T1. Number of responders over total enrolled patients is reported below each panel. TB, tuberculosis; TBI, tuberculosis infection; IMID, immune mediated inflammatory disease; TB1, peptides of TB1 tube of QFT-plus kit; TB2, peptides of TB2 tube of QFT-plus kit; IU/ml, international unit at millilitre; T0, before TB therapy, T1, after TB therapy completion. [file Image_1.tif]

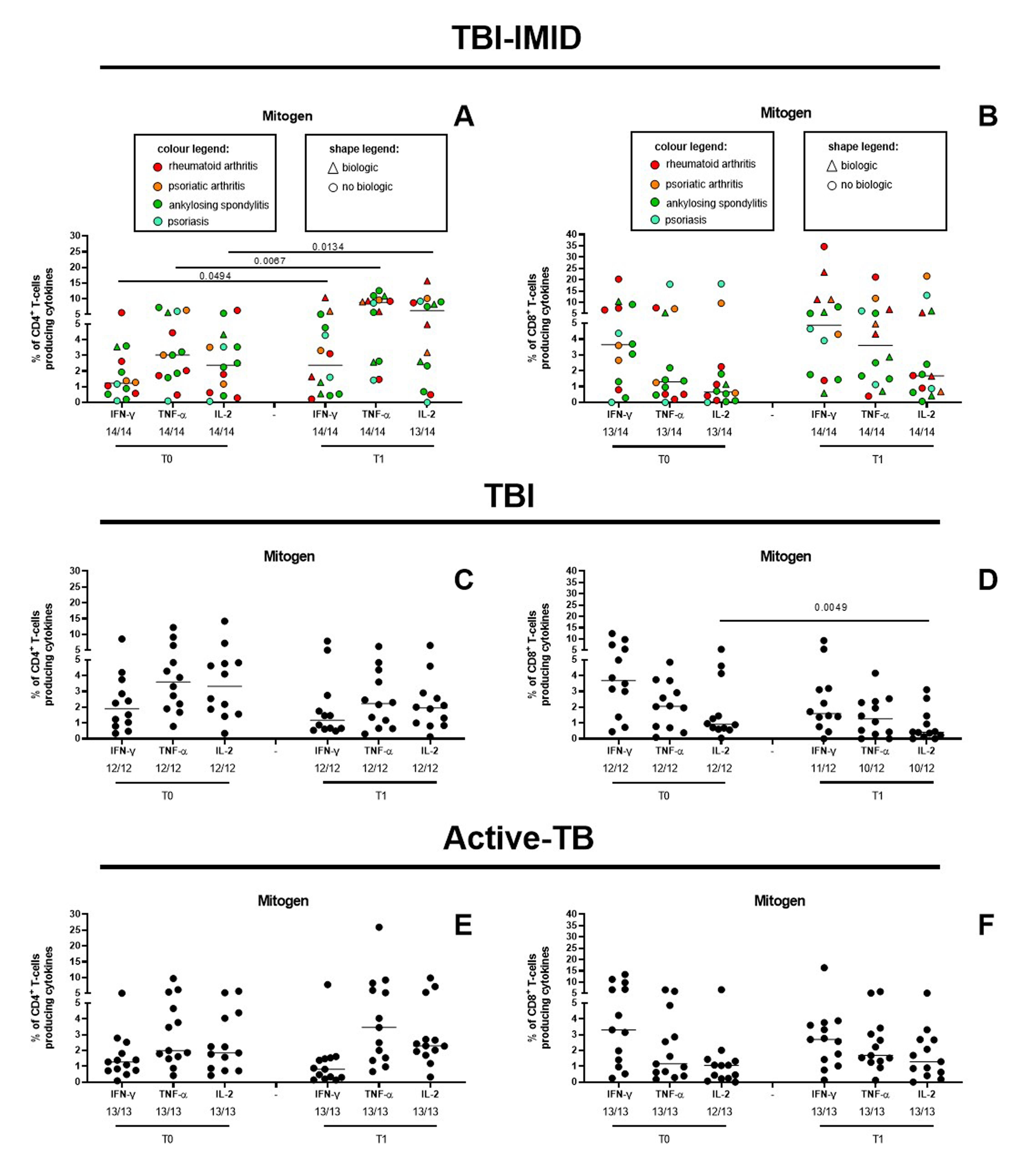

Supplement: Supplementary Figure 2 — Cytokine production CD4 and CD8 T cells in response to mitogen stimulation before and after TB therapy completion: PBMC of patients enrolled before (T0) and after TB therapy (T1) were stimulated overnight with Mitogen reagent of QFT-Plus kit. (A, B) TBI-IMID patients; (C, D) TBI subjects; (E, F) Active-TB patients. Number of responders over total enrolled patients is reported below each graph. Horizontal lines indicate the median. Statistical analysis was performed using the Wilcoxon matched-pairs signed rank test, and the p value was considered significant if ≤0.05 for cytokine production. TB, tuberculosis; TBI, tuberculosis infection; IMID, immune mediated inflammatory disease. [file Image_2.tif]

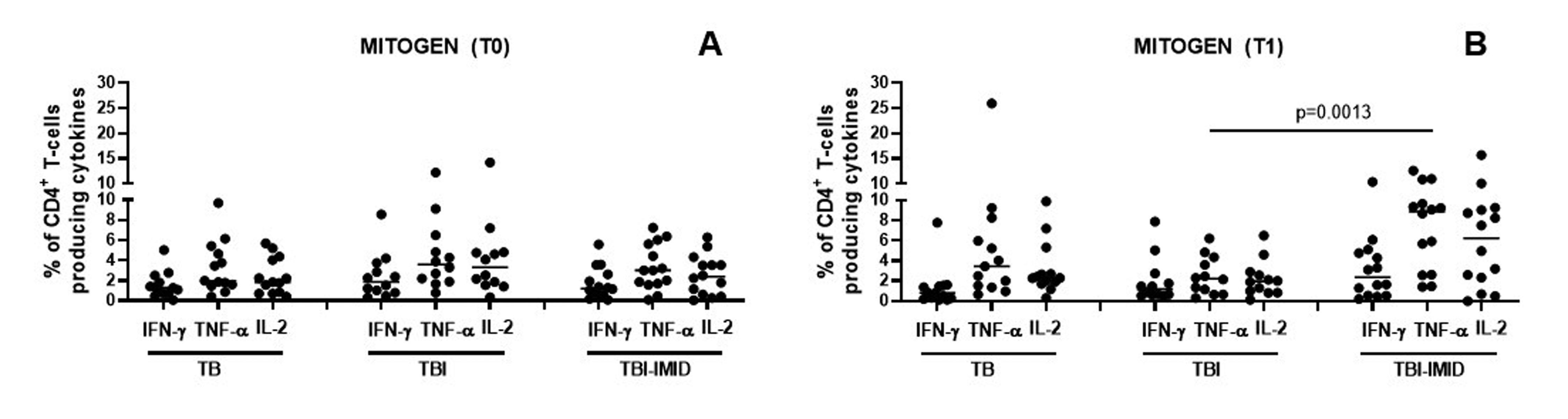

Supplement: Supplementary Figure 3 — Comparison of cytokine production CD4 and CD8 T cells in response to mitogen stimulation: PBMC of patients enrolled before (T0) and after TB therapy (T1) were stimulated overnight with Mitogen reagent of QFT-Plus kit. (A) T0; (B) T1. Number of responders over total enrolled patients is reported below each graph. Horizontal lines indicate the median. Statistical analysis was performed using the Mann–Whitney unmatched test, Bonferroni correction was applied, and the p value was considered significant if ≤0.017 for cytokine production. TB, tuberculosis; TBI, tuberculosis infection; IMID, immune mediated inflammatory disease. [file Image_3.tif]

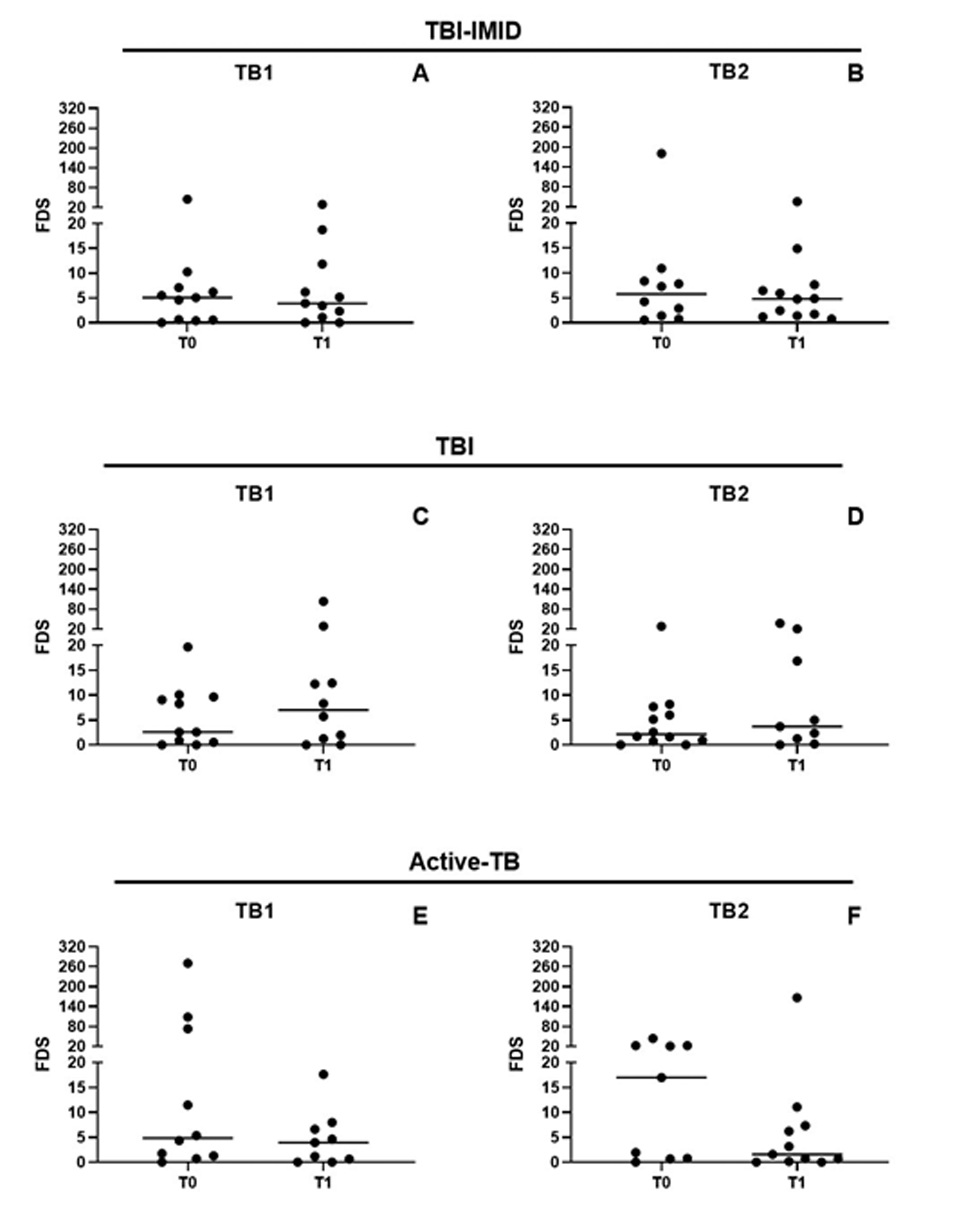

Supplement: Supplementary Figure 4 — Functional differentiation score (FDS) of Mtb-specific CD4 T cells is similar before and after TB therapy in TBI-IMID, TBI subjects, and active-TB patients. FDS has been calculated as previously described (28, 29) only on CD4 T-cell responders to Mtb antigen stimulation. (A, B) TBI-IMID patients; (C, D) TBI subjects; (E, F) Active-TB patients. Horizontal lines indicate the median. The number of CD4 and CD8 responders to TB1 and TB2 stimulation is reported in detail in Table 3. Statistical analysis was performed using the Wilcoxon matched-pairs signed rank test, and the p value was considered significant if ≤0.05. TB, tuberculosis; TBI, tuberculosis infection; IMID, immune mediated inflammatory disease; TB1, peptides of TB1 tube of QFT-plus kit; TB2, peptides of TB2 tube of QFT-plus kit; T0, before TB therapy; T1, after TB therapy completion. [file Image_4.tif]

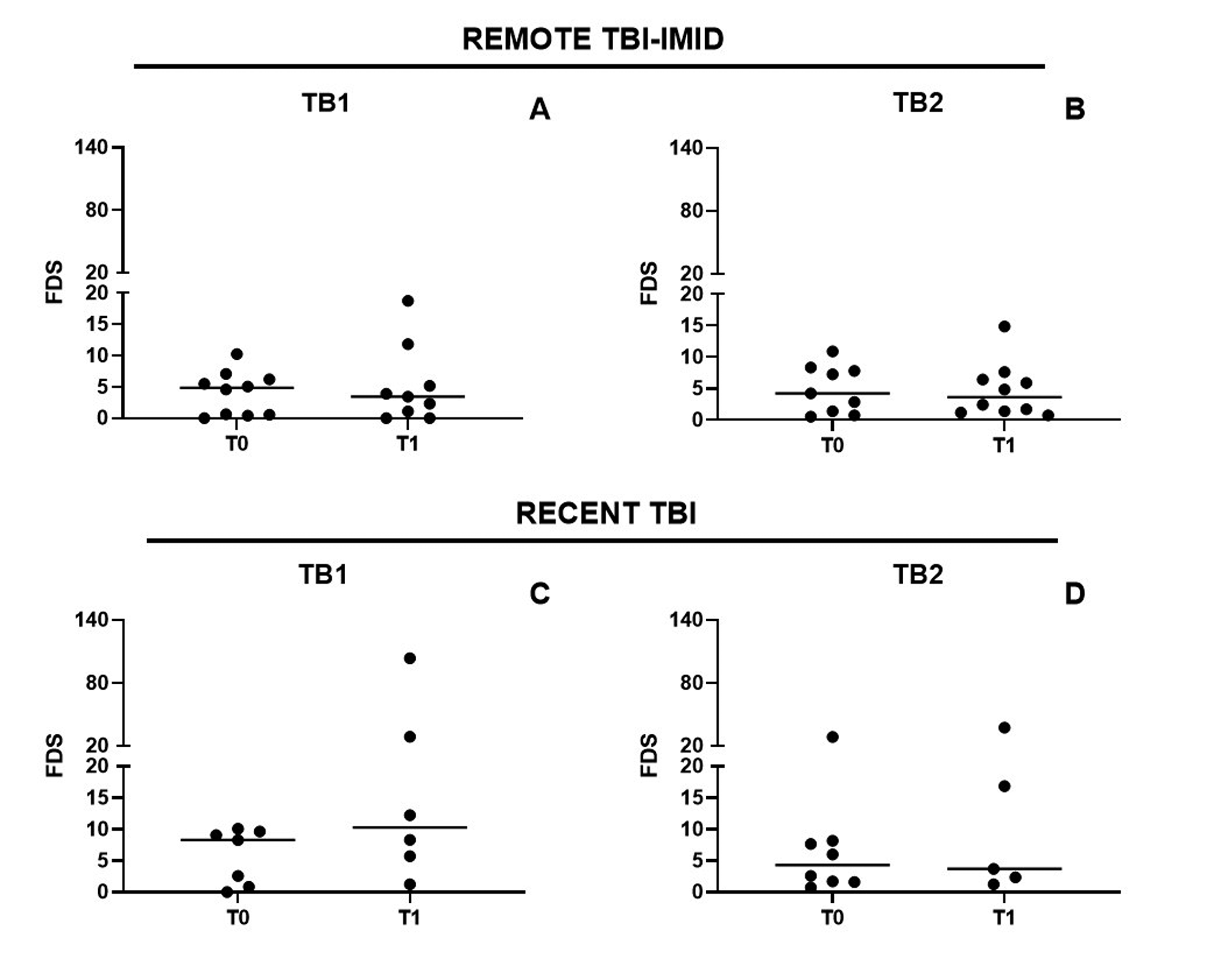

Supplement: Supplementary Figure 5 — Functional differentiation score (FDS) of Mtb-specific CD4 T cells is similar before and after TB therapy in TBI-IMID remote and TBI recent subjects. FDS has been calculated as previously described (28, 29) only on CD4 T-cell responders to Mtb antigen stimulation. (A, B) TBI-IMID remote patients, n = 12; (C, D) TBI recent subjects, N = 8. Horizontal lines indicate the median. Statistical analysis was performed using the Wilcoxon matched-pairs signed rank test, and the p value was considered significant if ≤0.05. TB, tuberculosis; TBI, tuberculosis infection; IMID, immune mediated inflammatory disease; TB1, peptides of TB1 tube of QFT-plus kit; TB2, peptides of TB2 tube of QFT-plus kit; T0, before TB therapy; T1, after TB therapy completion. [file Image_5.tif]
